# *In silico* study of principal sex hormone effects on post-injury synovial inflammatory response

**DOI:** 10.1371/journal.pone.0209582

**Published:** 2018-12-31

**Authors:** Bethany Powell, Igal Szleifer, Yasin Y. Dhaher

**Affiliations:** 1 Department of Biomedical Engineering, Northwestern University, Evanston, IL, United States of America; 2 Department of Mechanical Engineering and Bioengineering, Valparaiso University, Valparaiso, IN, United States of America; 3 Department of Chemistry, Northwestern University, Evanston, IL, United States of America; 4 Department of Chemical and Biological Engineering, Northwestern University, Evanston, IL, United States of America; 5 Department of Physical Medicine and Rehabilitation, University of Texas Southwestern Medical Center, Dallas, TX, United States of America; 6 Department of Orthopedic Surgery, University of Texas Southwestern Medical Center, Dallas, TX, United States of America; University of Texas Southwestern Medical Center, UNITED STATES

## Abstract

Following an anterior cruciate ligament injury, premenopausal females tend to experience poorer outcomes than males, and sex hormones are thought to contribute to the disparity. Evidence seems to suggest that the sex hormones estrogen, progesterone, and testosterone may regulate the inflammation caused by macrophages, which invade the knee after an injury. While the individual effects of hormones on macrophage inflammation have been studied in vitro, their combined effects on post-injury inflammation in the knee have not been examined, even though both males and females have detectable levels of both estrogen and testosterone. In the present work, we developed an *in silico* kinetic model of the post-injury inflammatory response in the human knee joint and the hormonal influences that may shape that response. Our results indicate that post-injury, sex hormone concentrations observed in females may lead to a more pro-inflammatory, catabolic environment, while the sex hormone concentrations observed in males may lead to a more anti-inflammatory environment. These findings suggest that the female hormonal milieu may lead to increased catabolism, potentially worsening post-injury damage to the cartilage for females compared to males. The model developed herein may inform future *in vitro* and *in vivo* studies that seek to uncover the origins of sex differences in outcomes and may ultimately serve as a starting point for developing targeted therapies to prevent or reduce the cartilage damage that results from post-injury inflammation, particularly for females.

## 1 Introduction

Females tend to have poorer prognoses after anterior cruciate ligament (ACL) injury compared to males, particularly with respect to cartilage damage [1, 2]. This difference has been widely observed, but few, if any, studies have attempted to uncover the biological link between sex and damage to the cartilage after knee injury. One potential link between sex and cartilage damage may be the inflammatory process, which is modulated by sex hormones and can modulate the production of matrix metalloproteinases (MMPs), the catabolic molecules that eventually cause permanent cartilage destruction [3]. However, while some studies have shown that hormones affect cytokine production [4–8] and other studies have shown that those cytokines affect production of MMPs [9–11], no study has comprehensively examined the effects of sex hormones on MMPs via their effects on inflammation in the synovial environment after ACL injury.

The principal sex hormones–estrogen, progesterone, and testosterone–each have distinct effects on macrophages, the primary invading cell type after ACL injury [12]. Testosterone, a predominantly male hormone, and progesterone, a predominantly female hormone, tend to promote a more anti-inflammatory response from macrophages [6, 7], while estrogen may be pro- or anti-inflammatory, depending on its concentration and microenvironment [13, 14]. In the synovial environment for males and pre-menopausal females, estrogen concentrations fall into a range where it has pro-inflammatory effects on macrophages [14, 15], though estrogen can have anti-inflammatory effects on macrophages at other concentrations or when acting on other cell types [14]. Together and individually, these three hormones have the potential to alter the inflammatory environment that develops after an ACL injury.

Although hormonal regulation of inflammation has not been directly connected to subsequent MMP production in the knee synovium, a clear connection has been established between inflammation and MMP production. Pro-inflammatory molecules like IL-1β and TNF-α enhance MMP production by multiple cell types, including macrophages and synovial fibroblasts (SFs), the resident cells of the synovium [9–11]. Furthermore, anti-inflammatory molecules like IL-10 can reduce MMP production [16], creating a complex environment with opposing effects of inflammatory mediators, along with hormonal action.

Such inflammatory environments have been examined *in silico* [17–19]. These models revealed insights about which cytokines exert the greatest influence on inflammation in a system of macrophages or a system with both macrophages and neutrophils, laying a methodological foundation for future *in silico* studies of inflammatory processes. However, these models could be adapted to include more thorough uncertainty analysis. One of the previous studies performed local sensitivity analysis to determine which cytokine would have the strongest effect on macrophage migration [19], but the study did not report how uncertainties in the nominal parameters would influence the time course of inflammation for all cytokines. Such analysis would help account for uncertainties in the *in vitro* experiments that were used to estimate the nominal values, such as varied experimental conditions and limited numbers of samples. Furthermore, such analysis would help account for biological differences that exist between the *in vitro* experiments from which the parameters were formulated and the *in vivo* states that the model sought to predict.

Previous models could also be adapted to include analysis of hormonal effects on the process of inflammation. To date, no model has incorporated the effects of sex hormones on the inflammatory responses under investigation.

Thus, in the present work, we sought to examine the interplay between sex hormones, inflammation, and MMP production in the injured synovium using an *in silico* approach. Recognizing the limitations of *in silico* studies, we accounted for uncertainties in the nominal model parameters using a statistical sampling approach that provided upper and lower bounds for our results. We hypothesized that 1) distinct patterns of inflammation would emerge when the cells found in the injured knee were exposed to expected male and female concentrations of the principal sex hormones, with a more pro-inflammatory response and greater MMP involvement for female concentrations, 2) the anti-inflammatory influence of progesterone would partially attenuate the pro-inflammatory influence of estrogen for females, and 3) the low estrogen concentrations at levels consistent with the early follicular phase would result in an attenuated inflammatory response compared to higher estrogen concentrations. With this quantitative framework, we aimed to shed light on the underlying processes that may cause increased cartilage damage for females after knee injury [20], and open avenues of research directed toward prevention or reduction of post-injury damage to the cartilage, particularly for females.

## 2 Methods

**Fig 1:**
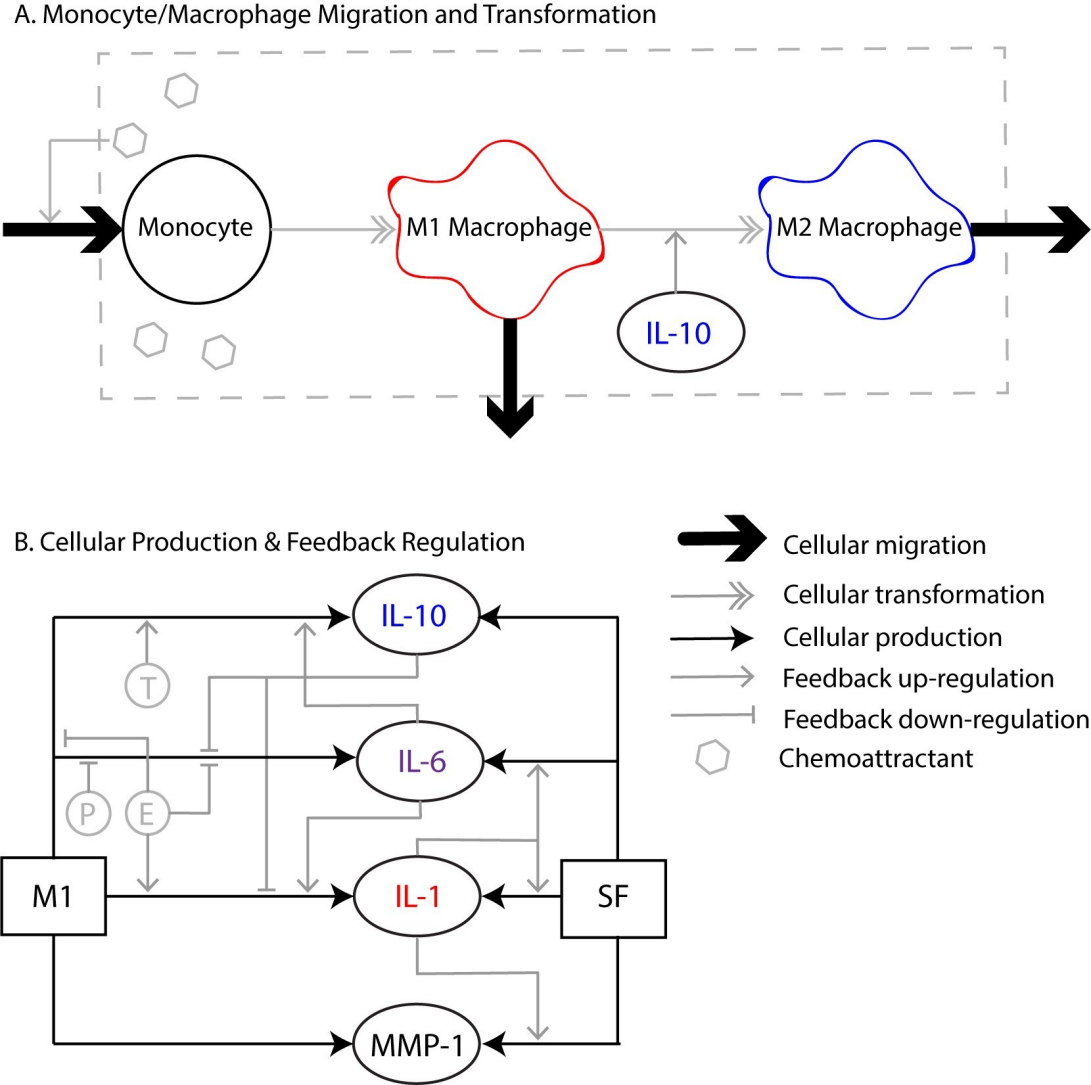
**A. Depiction of monocyte and macrophage migration and transformation.** Injury to the knee causes the production of chemoattractants, such as TNF-α and TGF-β, which lead to monocyte migration from the bloodstream to the synovium. The synovium is indicated by the gray dashed box. Monocytes transform into pro-inflammatory M1 macrophages. The molecular processes that drive transformation to M1 cells are not modeled. Instead, a 12-hour delay is incorporated into the model as a way to account for the time it takes for monocytes to transform, as previously described [19]. IL-10 drives the transformation of M1 cells to anti-inflammatory M2 macrophages [21]. Both M1 and M2 cells can migrate out of the synovium. **B. Cellular production and feedback regulation of a subset of the substances incorporated in the model.** M1 and SF both produce IL-10, IL-1, IL-6, and MMP-1. IL-10 down-regulates M1 production of IL-1 and IL-6, while IL-1 up-regulates SF production of IL-1, IL-6, and MMP-1. IL-6 up-regulates M1 IL-10 and up-regulates M1 IL-1. Estrogen (E) up-regulates M1 production of IL-1 and down-regulates M1 production of IL-10 and IL-6, while testosterone (T) up-regulates M1 production of IL-10. Progesterone (P) down-regulates M1 IL-6. M1: pro-inflammatory macrophage. M2: anti-inflammatory macrophage. SF: synovial fibroblast. E: estrogen. T: testosterone.

Using a system of first order differential equations, we modeled two key physiological processes: 1) cellular migration of monocytes/macrophages and platelets, and 2) cellular production of inflammatory mediators by those cells and by the resident synovial fibroblasts (SFs). Fig 1 shows these processes in a schematic form. The procedure for formulating such a model has been reported previously [19], but we also describe the procedure in detail here for completeness. To formulate the model parameters, we first performed an extensive review of literature to obtain *in vitro* studies that reported the quantitative data necessary for each parameter. This search included hundreds of search queries and resulted in over 40 useable publications from the PubMed database.

### 2.1 Production and decay

We calculated the production coefficients using the simplifying assumption that production was a linear function of time according to the expression:

$$k_{x,y}=\frac{C_{x}}{C_{y}t}$$ (1)

Where *C*_*x*_ is the concentration of the substance of interest in ng/mL, *C*_*y*_ is the experimentally reported concentration of cell type y in cells/mL, and *t* is the duration of cellular production in hours. To determine the degradation coefficients, we utilized experimentally reported half-lives for each substance:

$$k_{d,x}=\frac{0.693}{t_{1/2}}$$ (2)

where *t*_1/2_ is the half-life of a substance in hours. S1 Table shows all production coefficients and degradation coefficients, and S1 Text shows sample calculations for both quantities. In the sample calculations, we note the study from which the raw data were extracted, list the values obtained from the study, note the figure or table from which the data were extracted, and show the steps of the calculations. In total, we included 35 production and decay coefficients in our model, which we derived from 23 published reports.

### 2.2 Chemotaxis

Table 1 lists the equations for migration kinetics of macrophages and SFs. We assumed that SF concentration was constant throughout each simulation, while we allowed the macrophage concentrations to vary.

**Table 1:** Equations for cellular migration.

Model Variable	Initial Value	Concentration in Healthy Synovium	Equation
“Platelets”	$2*10^{8}\frac{cells}{mL}$	$0\frac{cells}{mL}$	$\frac{dC_{P}}{dt}=-k_{d,P}C_{P}$
M1 Macrophages	$0\frac{cells}{mL}$	$0\frac{cells}{mL}$	$\frac{dC_{M1}}{dt}=k_{M,in}(f_{TGF,M1}+f_{TNF,M1})-k_{M1M2}f_{M1M2}C_{M1}-k_{d,M}C_{M1}$
M2 Macrophages	$0\frac{cells}{mL}$	$0\frac{cells}{mL}$	$\frac{dC_{M2}}{dt}=k_{M1M2}f_{M1M2}C_{M1}-k_{d,M}C_{M2}$
SFs	$5*10^{5}\frac{cells}{mL}$		$\frac{dC_{SF}}{dt}=0$

Formulations of *f*_*TGF*,*M*1_ and *f*_*TNF*,*M*1_, and the values of their parameters can be found in S2 Table.

*In vitro* experiments have demonstrated that macrophages migrate in response to signals from TGF-β and TNF-α [22, 23]. Thus, we used chemotactic functions, *f*_*TGF*,*M*1_ and *f*_*TNF*,*M*1_, respectively, to incorporate migration into the present model. S2 Table lists the parameters for these functions. However, like a previous *in silico* study [19], we specified that these chemotactic functions would equal zero below a threshold platelet concentration (10 * 10^−12^ cells/mL) to prevent non-physiological re-initiation of inflammation. While this concentration is not rooted in physiology, it is nonetheless useful computationally and has been established as a technique to prevent non-physiological re-initiation of inflammation in the model [19].

### 2.3 Feedback modulation of concentrations

We used feedback functions to describe the changes in production of a given substance by other substances based on numerous cellular-level *in vitro* experiments, which we cite in S2 Table. In this procedure, we modeled negative feedback with the monotonically decreasing function

$$g_{j,x}=a*\exp\left(-b*C_{j}\right)+c$$ (3)

and we modeled positive feedback data with the monotonically increasing function

$$f_{j,x}=a*\frac{C_{j}}{1+C_{j}}$$ (4)

where *j* is the modulatory substance, *x* is the substance being modulated, *a*, *b*, and *c* are fitting parameters, and *C*_*j*_ is the concentration of the modulatory substance. We incorporated the up-regulatory functions found with Eq 4 into the model using the expression

$$g_{j,x}=1+f_{j,x},$$ (5)

to represent a fractional increase above the baseline production. S2 Table lists all the estimated parameters for chemotaxis functions and feedback functions. For clarity, we also included a detailed description of the process for generating feedback functions in the S1 Text. In total, we included 56 feedback coefficients in our model, which we derived from 24 published reports.

### 2.4 Time evolution of cytokine, MMP, and TIMP concentrations

We used the nominal production and decay coefficients that we found with Eqs 1 and 2, respectively, along with the feedback functions (Eqs 3–5) to model cellular production of cytokines, growth factors, and MMPs using the general form:

$$\frac{dC_{x}}{dt}=\left(\prod_{j}g_{j,x}\right)k_{x,M1}C_{M1}+k_{x,M2}C_{M2}+\left(\prod_{n}g_{n,x}\right)k_{x,SF}C_{SF}-k_{d,x}C_{x}$$ (6)

where *C*_*x*_ is the concentration of substance *x*, *k*_*x*,*M*1_ is the baseline production rate of substance *x* by M1 macrophages, *k*_*x*,*M*2_ is the production rate of substance *x* by M2 macrophages, *k*_*x*,*SF*_ is the baseline production rate of substance *x* by SFs, *C*_*M*1_ is the concentration of M1 macrophages, *C*_*M*2_ is the concentration of M2 macrophages, *C*_*SF*_ is the concentration of SFs, *k*_*d*,*x*_ is the degradation rate for substance *x*, *g*_*j*,*x*_ describes the feedback regulation of substance *x* by substance *j* in M1 macrophages, *g*_*n*,*x*_ describes the feedback regulation of substance *x* by substance *n* in SFs, and ∏_*j*_ is the product operator. Table 2 shows the specific equations for each substance using symbolic representation of the model parameters.

**Table 2:** Equations for cellular products.

Model Variable	Initial Value (from Model Steady State)	Concentration in Healthy Synovium	Ref.	Equation
IL-1β*	$0.037\frac{pg}{mL}$	$1\pm 2\frac{pg}{mL}$	[24]	$\frac{dC_{IL1}}{dt}=k_{IL1,M1}g_{IL10,IL1}g_{TGF,IL1}(1+f_{E2,IL1})(1+f_{IL6,IL1})C_{M1}+k_{IL1,M2}C_{M2}+k_{IL1,SF}(1+f_{TNF,IL1})(1+f_{IL1,IL1})C_{SF}-k_{d,IL1}C_{IL1}$
TNF-α	$0.155\frac{pg}{mL}$	$0\frac{pg}{mL}$	[24]	$\frac{dC_{TNF}}{dt}=k_{TNF,M1}g_{IL10,TNF}g_{TGF,TNF}{(1+f}_{IL6,TNF})g_{T,TNF}g_{PR,TNF}C_{M1}+k_{TNF,M2}C_{M2}+k_{TNF,SF}(1+f_{IL1,TNF})C_{SF}-k_{d,TNF}C_{TNF}$
IL-6*	$0.023\frac{pg}{mL}$	$64\pm 120\frac{pg}{mL}$	[24]	$\frac{dC_{IL6}}{dt}=k_{IL6,M1}g_{IL10,IL6}(1+f_{TGF,IL6})g_{E,IL6}g_{PR,IL6}C_{M1}+k_{IL6,M2}C_{M2}+k_{IL6,SF}(1+f_{IL1,IL6})(1+f_{TNF,IL6})C_{SF}-k_{d,IL6}C_{IL6}$
IL-10*	$0.073\frac{pg}{mL}$	$1\pm 6\frac{pg}{mL}$	[24]	$\frac{dC_{IL10}}{dt}=k_{IL10,M1}(1+f_{TGF,IL10})(1+f_{T,IL10})(1+f_{IL6,IL10})g_{E2,IL10}C_{M1}+k_{IL10,M2}C_{M2}+k_{IL10,SF}C_{SF}-k_{d,IL10}C_{IL10}$
TGF-β***	$5.684\frac{pg}{mL}$	$0\frac{pg}{mL}$	[25]	$\frac{dC_{TGF}}{dt}=k_{TGF,P}C_{P}+k_{TGF,M1}C_{M1}+k_{TGF,M2}C_{M2}+k_{TGF,SF}(1+f_{TNF,TGF})C_{SF}-k_{d,TGF}C_{TGF}$
TIMP-1	$2.42*10^{5}\frac{pg}{mL}$	$1.24*10^{5}\frac{pg}{mL}$	[26]	$\frac{dC_{TIMP}}{dt}=k_{TIMP,M1}g_{TNF,TIMPa}g_{IL1,TIMPa}g_{IL10,TIMP}C_{M1}+k_{TIMP,M2}C_{M2}+k_{TIMP,SF}(1+f_{TNF,TIMPb})(1+f_{IL1,TIMPb})(1+f_{IL6,TIMP})C_{SF}-k_{d,TIMP}C_{TIMP}$
MMP-9**	$0\frac{pg}{mL}$	$960\frac{pg}{mL}$	[27]	$\frac{dC_{MMP9}}{dt}=k_{MMP9,M1}(1+f_{IL1,MMP9})(1+f_{TNF,MMP9})g_{IL6,MMP9}C_{M1}+k_{MMP9,M2}C_{M2}-k_{d,MMP9}C_{MMP9}$
MMP-1	$1413\frac{pg}{mL}$	$3729\frac{pg}{mL}$	[26]	$\frac{dC_{MMP1}}{dt}=k_{MMP1,M1}C_{M1}+k_{MMP1,SF}(1+f_{TNF,MMP1})(1+f_{IL1,MMP1})C_{SF}-k_{d,MMP1}C_{MMP1}$

* In some cases, the margin of error for the measurements extended below a concentration of 0 pg/mL, suggesting that the experimental data may have been non-normally distributed, may have had extreme outliers, or may have been averaged over a small number of samples. However, very few studies report concentrations of cytokines in healthy joints, and the ones cited here serve as an adequate starting point for comparisons.

** Our estimate of initial MMP-9 concentration is lower than the concentration reported in a healthy knee. However, this will lead to a conservative estimate of its concentration once an inflammatory stimulus is included in the model.

*** Our estimate of TGF-β exceeds the concentration in a healthy joint. However, its initial value in the model is only about 1% of its peak value, so we argue that its effect will be minimal.

Here, we illustrate how we formulated the kinetic equations for IL-1β, relating the general form that we present in Eq 6 to the specific equations in Table 2. First, we found the coefficients that described baseline IL-1β production by M1, M2, and SFs using Eq 1 and data from published reports [28, 29], which we cited in S1 Table. This procedure led to numerical values for the coefficients *k*_*IL*1,*M*1_, *k*_*IL*1,*M*2_, and *k*_*IL*1,*SF*_, which were also listed in S1 Table. Second, we used data from another published report [30] (also cited in S1 Table) to formulate the decay coefficient, *k*_*d*,*IL*1_, using Eq 2. Third, we formulated the functions that describe feedback regulation of IL-1β production in M1 macrophages. In these cells, IL-1β is down regulated by IL-10 and TGF-β and up regulated by estrogen (E2) and IL-6 (see Fig 1). We used Eq 3 and previously reported *in vitro* data from two studies to estimate the feedback parameters for IL-10 and TGF-β down regulation of IL-1β in M1 macrophages [31, 32], leading to the expressions *g*_*IL*10,*IL*1_ and *g*_*TGF*,*IL*1_. We used Eq 4 and previously reported *in vitro* data from two other studies to estimate the feedback parameters for E2 and IL-6 up regulation of IL-1β [6, 33], leading to the expressions *f*_*E*2,*IL*1_ and *f*_*IL*6,*IL*1_, which we incorporate into Eq 5 to obtain the feedback up regulation functions *g*_*E*2,*IL*1_ = 1 + *f*_*E*2,*IL*1_ and *g*_*IL*6,*IL*1_ = 1+ *f*_*IL*6,*IL*1_. Fourth, we formulated the functions that describe feedback regulation of IL-1β production by SFs. In the case of IL-1β feedback regulation in SFs, all of the regulators are positive, so we use Eqs 4 and 5 and data from published *in vitro* studies to obtain the feedback functions *g*_*TNF*,*IL*1_ = 1+ *f*_*TNF*,*IL*1_, and *g*_*IL*1,*IL*1_ = 1+ *f*_*IL*1,*IL*1_ [29, 34]. These functions represented up regulation of SF IL-1β production by TNF-α and IL-1β, respectively. Finally, we combined terms to obtain the final equation for IL-1β concentration. We obtained the first additive term, *k*_*IL*1,*M*1_*g*_*IL*10,*IL*1_*g*_*TGF*,*IL*1_(1 + *f*_*E*2,*IL*1_)(1 + *f*_*IL*6,*IL*1_)*C*_*M*1_, by multiplying the M1 production coefficient for IL-1β (*k*_*IL*1,*M*1_) by all four M1 feedback functions (∏_*j*_*g*_*j*,*x*_) and the concentration of M1 macrophages (see S2 Table for a list of feedback functions and their parameters). We obtained the second additive term by multiplying the M2 IL-1β production coefficient (*k*_*IL*1,*M*2_) by the concentration of M2 macrophages (*C*_*M*2_). We obtained the third additive term by multiplying the SF production coefficient for IL-1β (*k*_*IL*1,*SF*_) by the SF feedback functions (∏_*n*_*g*_*n*,*x*_) and the concentration of SFs, *C*_*SF*_, leading to the term *k*_*IL*1,*SF*_(1 + *f*_*TNF*,*IL*1_)(1 + *f*_*IL*1,*IL*1_)*C*_*SF*_. And we obtained the final additive term by multiplying the negative of the decay coefficient for IL-1β (−*k*_*d*,*IL*1_) by the present concentration of IL-1β (*C*_*IL*1_), leading to the term −*k*_*d*,*IL*1_*C*_*IL*1_. Summing all of these additive terms led to the expression for the change in concentration of IL-1β listed in Table 2 and follows from Eqs 1–6. We used this same procedure to find the rest of the equations in Table 2 and listed the resulting parameters and feedback functions in S1 and S2 Tables.

To summarize our approach for formulating the equations that describe time evolution of concentration, we note that there are four key calculations required to formulate the parameters for each substance in the model: 1) calculation of the production coefficients, 2) calculation of the degradation coefficients, 3) calculation of feedback down-regulation functions, and 4) calculation of feedback up-regulation functions. To clarify this procedure and facilitate reproducibility, we demonstrate an example of each of these calculations in S1 Text, which also includes the citations for the studies we used to estimate the parameters. Using the parameters calculated with these steps, we implement the differential equations in an open-source code (S1 Scripts).

### 2.5 Determination of initial conditions

The first step toward understanding the cascade of inflammation after an injury is understanding the initial conditions in the healthy joint before injury has occurred. We attempted to reflect the cellular environment of a healthy knee with our model by running a preliminary simulation in which we only allowed the SFs to produce cytokines in the absence of macrophages. We then used the steady-state concentrations that resulted from this simulation as the initial conditions for subsequent simulations where we included macrophages. This technique for finding initial conditions has been described previously [35].

SF concentration in a healthy joint is not well defined. Therefore, for lack of better information, we used a typical experimental *in vitro* cellular concentration of SFs, 5 * 10^5^ cells/mL [36]. Additionally, we used a platelet concentration of 2 * 10^8^ platelets/mL to initiate the inflammation [19].

### 2.6 Probabilistic modeling and analysis

**Fig 2:**
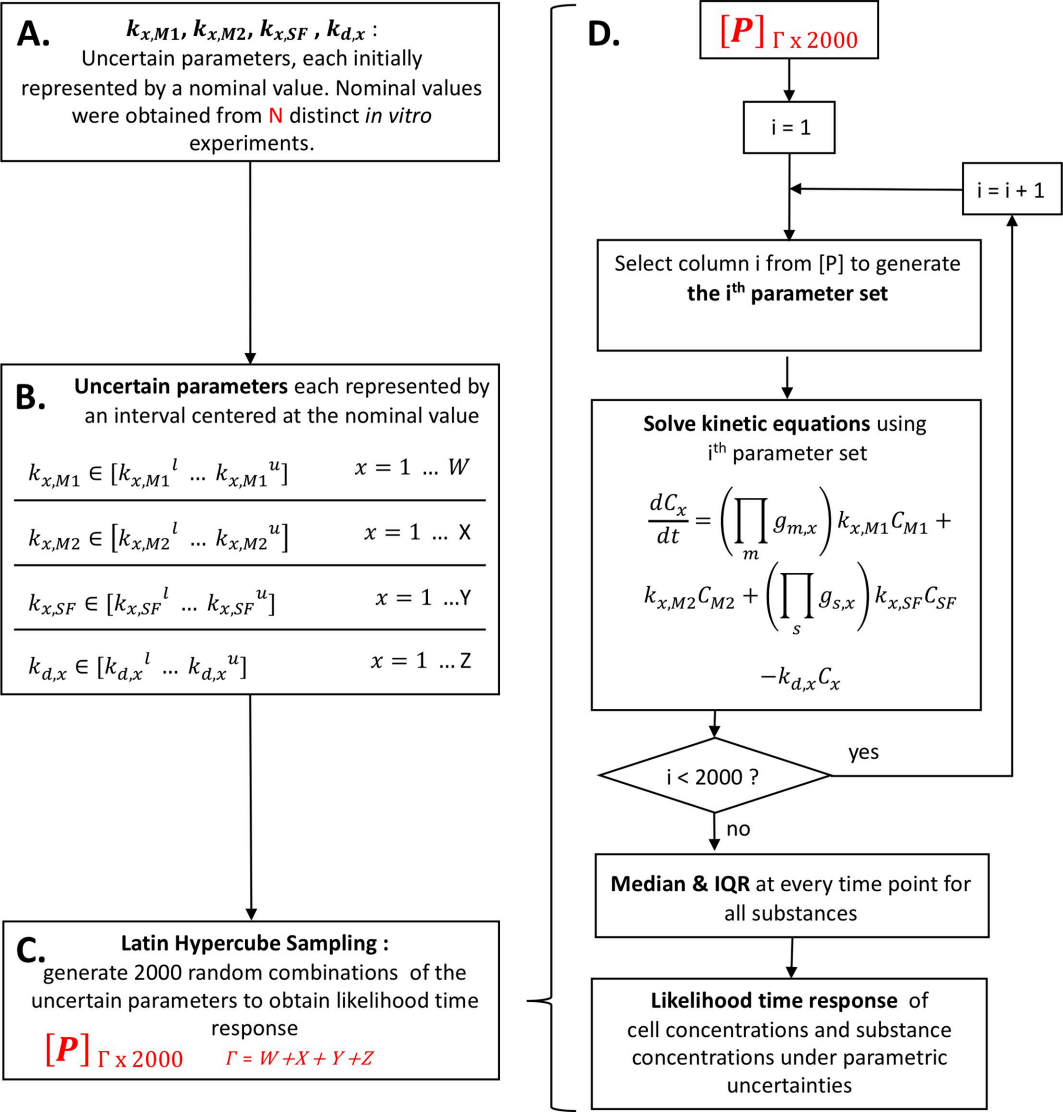
Summary of model formulation process. **A. Estimation of Nominal Parameter Values.** The first step in the modeling process was to estimate the nominal production and decay rate coefficients (see section 2.1) from N = 23 published *in vitro* studies (cited in S1 Table). **B. Parametric Variations.** We varied these parameters between 40% and 160% (±60%) of their nominal values, generating a 2000 element row vector associated with every parameter. The values in these row vectors were evenly spaced and sorted in ascending order. **C. Parameter Matrix [P].** Next, we randomized the order of each individual row vector before stacking the vectors into a Γ by 2000 matrix, [P], where Γ represented the number of nominal parameters (and, therefore, the number of row vectors) in the model. Because of the randomization and stacking, each individual column in [P] represented a randomly varied parameter set that we could use in the differential equations. **D. Latin Hypercube Sampling Process.** We selected column i from [P] to generate the ith parameter set and solved the differential equations. After solving the equations with all 2000 randomly varied parameter sets, we determined the median and interquartile range of the results at every time point for every substance, generating the likelihood time responses for concentration under parametric uncertainties. This parametric uncertainty analysis helped us account for uncertainties in the *in vitro* experiments that we used to estimate the nominal values, such as varied experimental conditions and limited numbers of samples. Furthermore, this analysis helped us account for biological differences that exist between the *in vitro* experiments from which we formulated the parameters and the *in vivo* states that we sought to model.

We used Latin Hypercube Sampling, a statistical sensitivity analysis method commonly used in computer simulation applications, to account for uncertainty in the nominal parameters that we found using Eqs 1–5. With Latin Hypercube Sampling, we randomly varied each production and decay coefficient within a range of ±60% of its nominal value (S1 Table), generating 2000 unique parameter sets and 2000 corresponding solutions of the differential equations. We next determined the median and interquartile range for each respective cytokine at every time point to generate margins of error for our results. Fig 2 schematically depicts this statistical sampling technique. We generated these margins of error in the absence of hormones to facilitate comparisons with independent *in vivo* data that did not account for hormonal effects [37].

We then performed two analyses of hormonal effects on the post-injury inflammatory response using concentrations reported in Greenspan *et al*. [38]. First, we used the nominal parameter set to examine the effects of isolated hormones at single concentrations using three scenarios: 1) estrogen alone (143 pM); 2) estrogen plus progesterone (143 pM and 990 pM, respectively); and 3) testosterone alone (20 nM). Second, after examining the effects of isolated hormone concentrations at single concentrations with the nominal parameter set, we performed simulations with combined estrogen and testosterone with ranges of possible concentrations, since males have non-negligible levels of circulating estrogen and females have detectable concentrations of testosterone in the blood [38]. Using Latin Hypercube Sampling, we varied estrogen and testosterone within physiological ranges for both females and males and simultaneously varied the model coefficients in a balanced fashion, varying the coefficients by ±20% in this analysis. We included two female conditions: 1) “Female Peak E” with low testosterone concentrations and the highest 20% of estrogen concentrations observed during the female menstrual cycle and 2) “Female Low E” with low testosterone concentrations and the lowest 20% of estrogen concentrations observed during the cycle. In addition, we incorporated a condition with high testosterone and low estrogen to represent male concentrations (“Male”). Table 3 shows these conditions and the corresponding hormone ranges.

**Table 3:** Estrogen and testosterone ranges for males and females. These concentrations were obtained from Greenspan et al. (2004) [38].

	Minimum E (pM)	Maximum E (pM)	Minimum T (nM)	Maximum T (nM)
Male	1.0	106	8.7	38.1
Female Low E	143	673	0.069	1.38
Female Peak E	2266	2797	0.069	1.38

### 2.7 *In vivo* verification literature

We reviewed the literature to obtain *in vivo* studies that examined the time-course of inflammation in the knee joint after ACL injury (the state we sought to examine) by searching the PubMed database, using the “Best Match” and “Most Recent” features. For each substance, we searched keywords such as “cytokine concentration synovial fluid,” “MMP concentration synovial fluid,” and “in vivo synovial cytokine concentration,” where the word “cytokine” could be replaced with the name of any individual cytokine. We combined these terms with “ACL injury” and “healthy” to obtain studies of synovial cytokine concentration in the states that we simulated with the model.

### 2.8 Statistical analysis

We used Kruskal-Wallis to test for differences between “Female Peak E,” “Female Low E,” and “Male” at a t = 1 day and Mann-Whitney U testing with the Bonferroni correction to do pairwise hypothesis testing *post hoc*. We then repeated these tests at the start of each subsequent day up to t = 20 days using the MATLAB R2017b Statistics and Machine Learning Toolbox. S1 Scripts contains the .m file for this statistical analysis.

## 3 Results

### 3.1 Initial conditions

Table 2 (“Initial Value (from Model Steady State)” column) shows the initial conditions that resulted from running the simulation in the absence of an inflammatory stimulus (SFs only, no macrophages) and the column entitled, “Concentration in Healthy Synovium,” shows the *in vivo* concentrations of these substances in healthy knee joints. Our data appear to capture the same order of magnitude as experimentally measured quantities in the cases of TIMP-1, MMP-1, and TNF-α. Our data also appear to be in the experimentally reported confidence intervals in the cases of IL-1β, IL-6, and IL-10. In the cases of TGF-β and MMP-9, our initial conditions do not appear to show close agreement.

### 3.2 Verification

**Fig 3:**
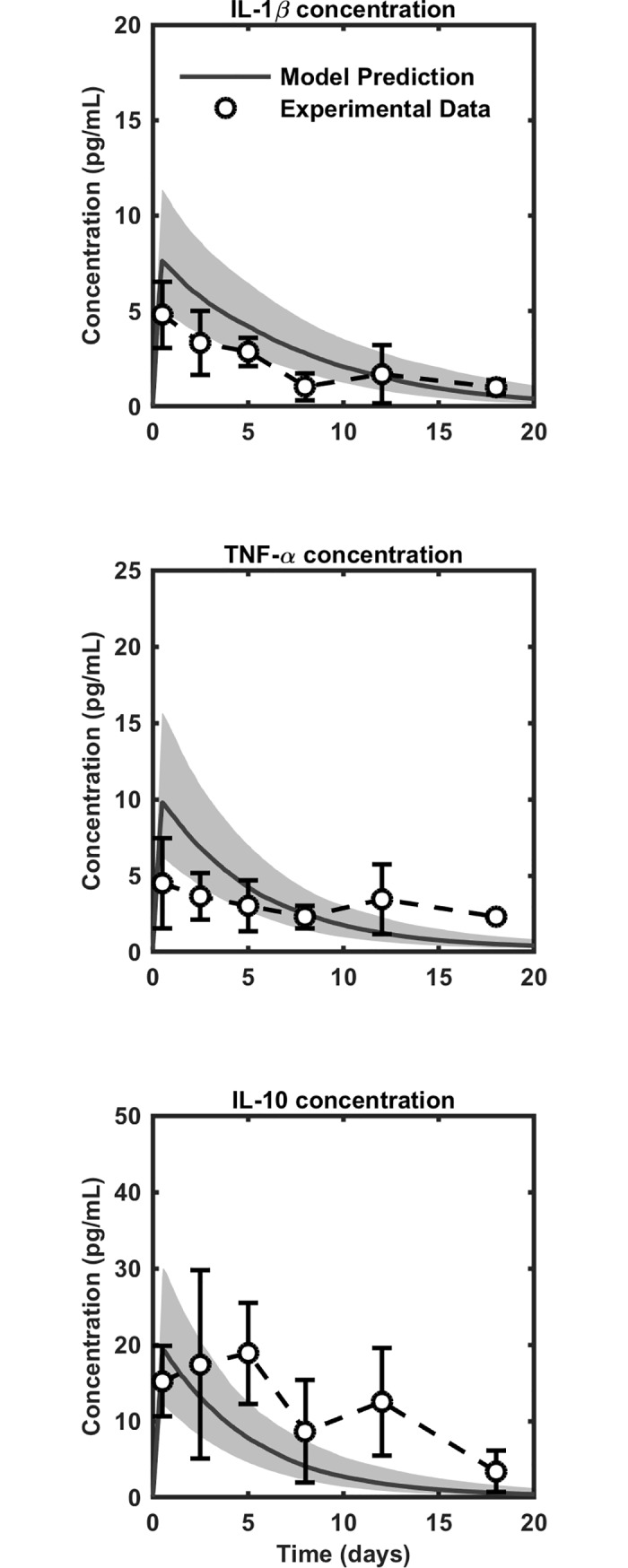
Model results for latin hypercube sampling analysis of IL-1β, TNF-α, and IL-10 compared to independent *in vivo* synovial concentrations following anterior cruciate ligament (ACL) injury [37]. Simulation results shown as median (solid gray lines) ± IQR (gray bands). In vivo comparison data are shown as circles with error bars. See S1 Fig and S4 Table for independent comparisons of the rest of the substances in the model.

The cytokine concentrations predicted by our model appear to be qualitatively consistent with independent *in vivo* experimental results [37] (Fig 3; S1 Fig and S4 Table). Fig 3 shows that independent *in vivo* cytokine concentration data (that is, data not used for parameter estimation or model formulation) appear to confirm the salient features of the bottom-up model output: a peak concentration that occurs shortly after injury and a gradual decrease in concentration over 20 days. Further, we note that the bottom-up model results and the experimental values show approximate numerical agreement at most time points in Fig 3. Unfortunately, only one independent *in vivo* study reported the time-course of synovial cytokine concentration after ACL injury *in vivo* and that study did not include all the substances modeled here [37]. S1 Fig shows the time-course of concentrations for the substances that were not included in [37]. However, while *in vivo* time-course data do not exist for some of the substances in the model, *in vivo* data for single time points after injury have been reported for the remaining substances. We include these data in S4 Table. The data in S4 Table appear to show reasonable order-of-magnitude agreement with the concentrations of cytokines, MMPs, and TIMP-1 in S1 Fig.

### 3.3 Hormonal considerations

**Fig 4:**
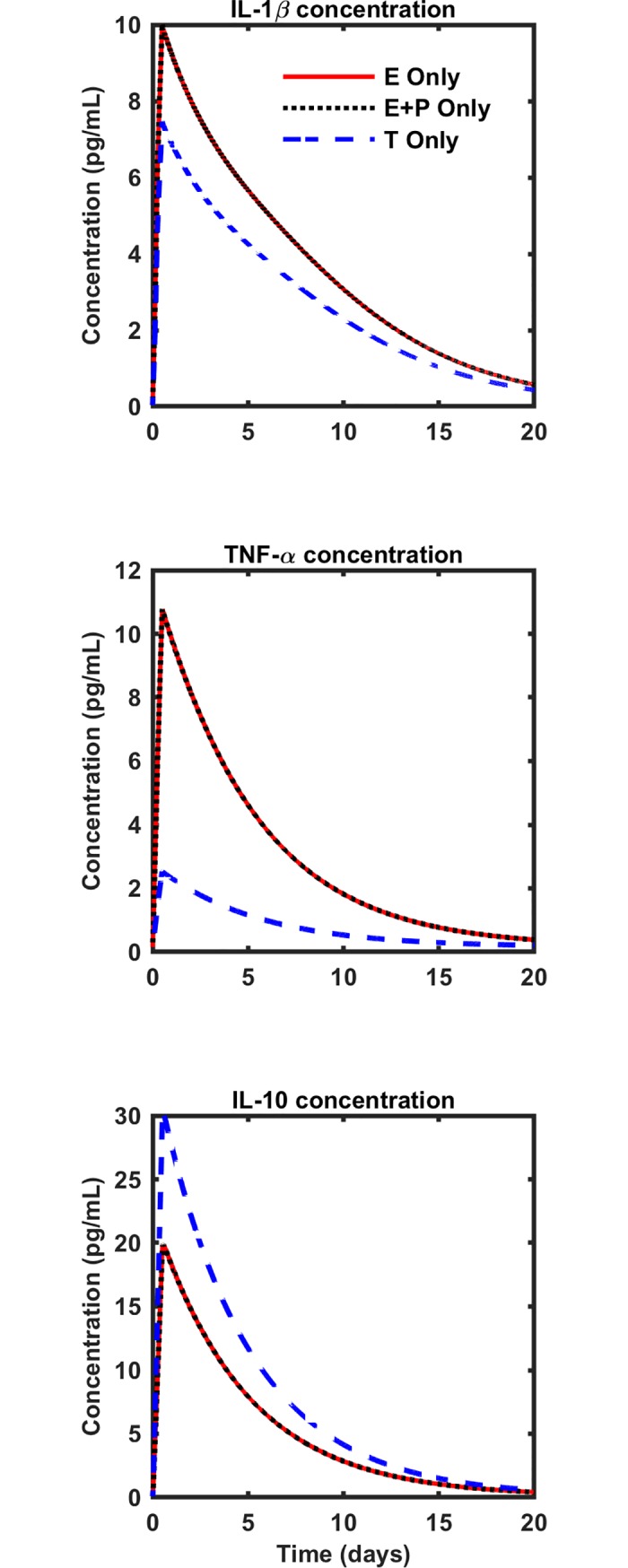
Using the nominal parameter set, sex hormones modulate post-injury IL-1β, TNF-α, and IL-10. E Only: low estrogen concentration, as for the early follicular phase (143 pM); E + P: low levels of estrogen and progesterone, as for the early follicular phase (143 pM estrogen, 990 pM progesterone); T Only: testosterone concentration in the normal range for an adult male (20 nM).

Fig 4 demonstrates that isolated estrogen leads to higher concentrations of IL-1β and TNF-α, as well as lower IL-10 concentration compared with male levels of isolated testosterone. Progesterone does not appear to attenuate the pro-inflammatory effects of estrogen at a physiologically relevant concentration.

**Fig 5:**
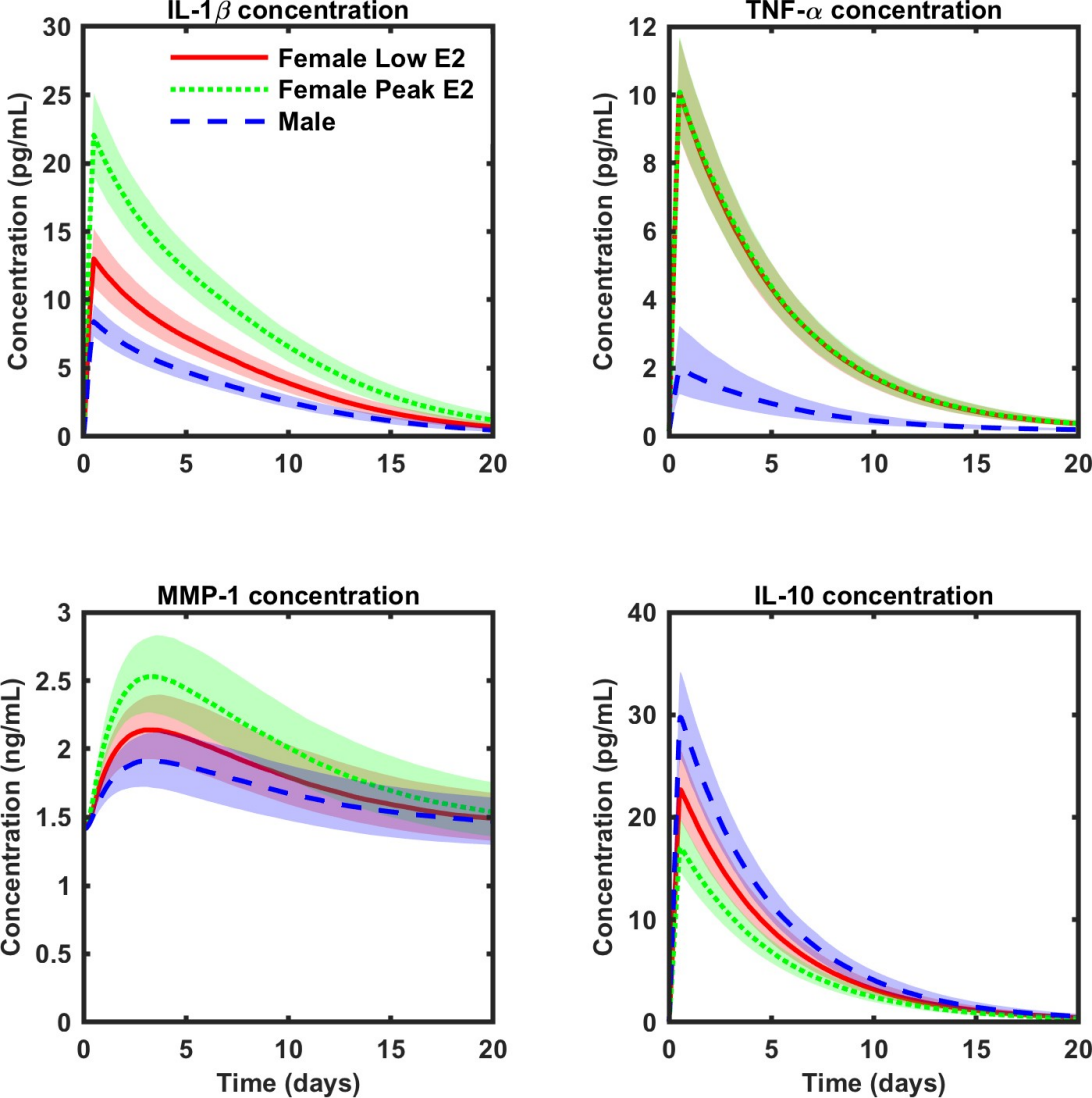
Effects of combined estrogen and testosterone at physiological levels for males and females (median ± IQR). Blue: combined T and E at concentrations for adult males; red solid: females with combined T and E at concentrations for females in the early follicular phase (Female Low E); green dotted: combined T and E for females with a steady estrogen concentration that varies around the peak value of estrogen during the menstrual cycle (Female Peak E). Kruskal-Wallis testing and *post hoc* Mann-Whitney U testing (with the Bonferroni correction) reveal highly significant differences between hormonal conditions at nearly all time points for all substances (analysis included in S1 Scripts). For IL-1β, IL-10, and MMP-1, there are significant differences between every possible hormone pair: “Male” is significantly different from “Female Peak E;” “Male” is significantly different from “Female Low E;” and “Female Peak E” is significantly different from “Female Low E.” The difference in MMP-1 concentration between “Male” and “Female Low E” lose significance at t = 19 days and t = 20 days. TNF-α is the only exception, as it shows no significant differences between “Female Low E” and “Female Peak E” at any time point. Further, no differences exist between hormone conditions t = 0 days, since the initial conditions are the same, regardless of the hormone treatment.

Fig 5 shows the median and interquartile range for cytokine concentrations when the model parameters and sex hormone concentrations are varied simultaneously. The “Female Peak E” condition leads to significant elevation of IL-1β and MMP-1, and significant suppression of IL-10 compared to the “Male" condition. The “Female Low E” condition also appears to elevate IL-1β and MMP-1 and suppress IL-10 compared to the “Male” condition, but to a lesser degree than the “Female Peak E” condition. Nonetheless, the differences between “Female Low E” and “Male” remain significant through the duration of the simulation for IL-1β and IL-10 and until t = 19 days for MMP-1. Both “Female Peak E” and “Female Low E” lead to significantly elevated TNF-α concentration compared to the “Male” condition throughout the time frame of the analysis. Conversely, TNF-α concentration did not significantly differ between “Female Peak E” and “Female Low E” at any time point.

## 4 Discussion

### 4.1 Summary of findings

In the present work, we examined the effects of the three principal sex hormones on inflammation and MMP production by macrophages and SFs using a systems biology approach. First, we formulated the model in the absence of hormonal effects, using data from more than 40 published reports of *in vitro* cellular studies to model the cellular and molecular processes that occur in concert in the knee joint after injury. After estimating the nominal parameters from these *in vitro* studies, we performed Latin Hypercube sampling to account for uncertainties in our estimated parameters, generating 2000 model solutions. We used the results from those 2000 statistically varied solutions to compare our model results to independent experimental data *post hoc*. For this comparison, we found that our results captured the salient features of data from the only available *in vivo* study that reported the time course of cytokine concentration in the synovium after an acute ACL injury [37] for a select subset of substances in our model (Fig 3). Further, we found that the rest of the substances in our model appear to agree with concentration data from single time points after ACL injury (S1 Fig and S4 Table).

Following formulation, statistical variation, and comparison to independent experimental data, we used the model to investigate the effects of single concentrations of estrogen, estrogen plus progesterone, and testosterone on the inflammatory response. Our simulation results indicated that estrogen led to a pro-inflammatory effect, testosterone led to an anti-inflammatory effect, and progesterone had little influence on the post-injury inflammatory response. However, because males and females have non-negligible levels of both estrogen and testosterone, we also used Latin Hypercube Sampling to generate a range of solutions that could result from typical hormone concentrations. We found that female combinations of estrogen and testosterone (“Female Low E” and “Female Peak E” conditions) both led to higher concentrations of pro-inflammatory IL-1β and TNF-α and lower concentrations of IL-10 compared to the “Male” condition. Further, model simulations indicated that the “Female Peak E” condition led to increased MMP-1 production compared with the “Male” condition. Together, these results suggest that estrogen, which varies in concentration through the menstrual cycle, has the potential to affect the course of the inflammatory process after knee injury. To our knowledge, this is the first model that can be used as a platform to study hormonal influences on post-injury inflammation while addressing some of the biological complexity of the post-injury synovium.

### 4.2 Model assumptions

Our systems biology approach is consistent with other *in silico* studies of inflammatory processes in other model systems [17–19]. As such, the approach relies on estimated rate coefficients and feedback parameters, which we determined based on simplifying assumptions and limited data from numerous independent *in vitro* experiments. One simplifying assumption is that the effects seen in isolated *in vitro* experiments will not qualitatively change as the environment becomes more complex. For example, we assume that a parameter for uptake of one substance does not change in the presence of another substance, and that the functional forms of feedback regulation do not change as the environment changes. These assumptions make it tractable to study the inflammatory response when multiple cell types and multiple hormones are present, since it is experimentally complex, even intractable, to conduct multi-variate *in vitro* experiments that emulate the real biological state of the injured knee joint. Thus, we argue that the present model represents a first step toward understanding post-injury knee inflammation at the systems level that would otherwise be quite difficult to examine experimentally.

In our model of the acutely ACL-injured knee joint, we included a number of cytokines, chemokines, and growth factors that contribute to sex hormonal regulation of MMPs [6–8, 37, 39], while we chose to exclude other cytokines and molecules implicated in joint inflammation. For example, we did not include IL-8 (also known as CXCL8), even though, in some cases, it has been detected in the knee joint after injury [39]. Traditionally, IL-8 acts primarily as a chemoattractant for neutrophils [40], but evidence suggests neutrophils do not infiltrate the knee joint after ACL injury and during OA [12, 41–44]. Thus, the exact purpose of IL-8 in the ACL-injured synovium is unclear, making unclear why IL-8 has been detected in the joint after injury and making it difficult to justify its inclusion in our model. Furthermore, we chose to exclude damage associated molecular patterns (DAMPs) from our analysis, even though they can play an important role in the joint after ACL injury and during the initiation and progression of OA. DAMPs, which can result from tissue damage, can activate toll-like receptors (TLRs) and perpetuate inflammation in the long term [45]. Unfortunately, not enough quantitative data exist to allow us to formulate parameters that describe this process, so we were unable to include DAMPs in the present model.

In the present model, we included “platelets” as a tool to initiate the inflammatory response because the cellular and molecular initiators of the inflammatory response after joint injury are not well understood [46]. However, we did not investigate clotting nor other effects of platelets. We specified that the platelets would release TGF-β, then rapidly decrease in concentration to negligible levels within hours of the onset of inflammation. Those cells were labeled as platelets because the model parameters for the release of TGF-β, an important driver of macrophage chemotaxis [23], were derived from experiments with platelets [47, 48]. Further, we note that the inclusion of platelets as a trigger to inflammation has also been reported by other investigators [19].

To account for experimentally observed effects of sex hormones on inflammation, we incorporated specific hormonal feedback functions in the model and used the framework described in the methodology. We assumed that synovial concentrations were equal to serum concentrations of hormones, since no significant difference exists between serum and synovial concentrations [15]. We also assumed that estrogen was pro-inflammatory, and that progesterone and testosterone were anti-inflammatory in the environment of the injured knee based on published data from cellular level experiments [4–8].

### 4.3 Model contextualization

We selected estrogen concentrations to match the concentration of freely circulating estrogen in non-pregnant, pre-menopausal females, and those concentrations were within the range where the hormone produces a pro-inflammatory response [14]. The relationship between macrophage production of IL-1β and estrogen appears to be non-monotonic, leading to a pro-inflammatory effect at menopausal to peri-ovulatory concentrations, but a suppressive effect at pregnancy concentrations [14]. That is, estrogen may be pro- or anti-inflammatory, depending on the cell types and estrogen concentrations present. Several factors may contribute to these discrepancies, including method of macrophage polarization, relative expressions of estrogen receptor (ER)-α and ER-β, and estrogen dosage. However, for the cell types involved in post-injury joint inflammation and the estrogen concentrations in the synovium for men and non-pregnant pre-menopausal women, estrogen is likely pro-inflammatory.

Additionally, the present work only addresses hormonal effects associated with pre-menopausal females that do not use exogenous hormones (hormonal contraceptives). We narrowed the focus of this study to this group for several reasons. First, post-menopausal females appear to have increased risk of osteoarthritis for reasons that are not completely understood [49], and those risk factors may affect the inflammatory response under investigation. Second, formulations of hormonal contraceptives vary widely, particularly for the progestins. Some formulations of progestin have androgenic effects while others appear to have anti-androgenic effects [50]. In either case, the research community does not quantitatively understand the effects of the synthetic progestins on macrophage and SF production of cytokines and MMPs well enough to allow a thorough, model-based synthesis. Finally, we did not examine the effects of hormonal fluctuations associated with the menstrual cycle, though these effects are the subject of ongoing work. Such investigations are beyond the scope of the present work, which sought to establish a model to examine hormonal effects and examine potential differences in the male and female inflammatory response in the synovium after ACL injury.

Emerging research seems to indicate that hormonal effects *in vitro* may not only depend on the hormones and cell types present, but also may depend on the sex of the donor of the cells; the cellular responses will differ for XX cells compared to XY cells [51]. The results of these studies represent an important step in our understanding of cellular behavior and we should ultimately incorporate them into multi-factorial kinetic models like the present model. However, we could not include the effect of cell sex in this study because most *in vitro* studies give no clear classification of the sex of the animal or human cell donor (see S1 and S2 Tables; n.r. indicates that sex was not reported in the cited study). Nonetheless, the present model represents an important first step toward understanding hormonal contributions to the inflammation that occurs in the knee synovium after an ACL injury, and may serve as a building block for future work that can also account for sex differences in the cellular responses.

The model may also serve as a building block that could help inform the design of future *in vivo* or *in vitro* experiments by identifying the relative contributions of hormones, cells, cytokines, and time analytically. When designing experimental studies of cytokines, it can be difficult to pinpoint which cytokines are most important to investigate, and experimental costs can rise quickly if too many cytokines are investigated. To narrow the scope of such experiments, *in silico* models such as this one could be used to help determine which subset of cytokines will be most critical to the *in vivo* and *in vitro* processes of interest.

### 4.4 Verification considerations

Running the model in the absence of an inflammatory stimulus, we found that most our predictions agreed reasonably with experimentally reported concentrations in healthy knees (Table 2). However, we note that the experimental confidence intervals for IL-1β, IL-6, and IL-10 extended below zero; that is, the measured error was larger than the mean value of the measurements. For example, the mean concentration of IL-6 was 64 pg/mL while its standard deviation was 120 pg/mL. Such large errors may be a result of non-normally distributed data or small sample sizes. However, despite large variability, these data serve as important points of comparison because few studies report the concentrations of synovial cytokines in healthy knee joints.

We found that our estimate of MMP-9 in the absence of inflammation did not appear to agree with experimental measurements from healthy knees. This discrepancy may have arisen either because our model did not incorporate all possible cell types in the healthy joint or because of experimental uncertainties in the study that reported MMP-9 concentration in the healthy joint. In our model, we only incorporated SFs during the simulation of the healthy joint and did not include potential effects of the resident macrophages during this particular analysis. While the resident macrophages are not polarized toward an inflammatory phenotype in the healthy joint [52], it is possible that they may produce substances that the SFs do not, such as MMP-9. In that case, our model would not have been able to capture the concentration of MMP-9 in the healthy joint because it did not include the synovial macrophages in the healthy state. However, experimental uncertainties may have also influenced the MMP-9 measurement, causing experimental concentrations to disagree with the model predictions. For example, the study that measured MMP-9 might have left variables uncontrolled that had the potential to influence cytokine and MMP-9 concentrations. Such variables may include undetected sub-acute joint inflammation, activity levels on the day of testing, or use of external stimuli like caffeine or alcohol [53–55]. However, it is not entirely clear whether experimental uncertainties or model shortcomings were responsible for the observed discrepancy in MMP-9 concentration in the healthy joint.

Our estimate of TGF-β concentration in the healthy state also differed from experimental measurements of its concentration in healthy knees. However, we argue that this discrepancy between concentrations in the healthy state is unimportant in the context of the inflammatory process, since the discrepancy of 5.7 pg/mL is only about 1% of the peak concentration of TGF-β. Indeed, despite some discrepancies between our estimates and experimental data in the healthy state, the model appears to appropriately capture important aspects of experimentally measured data when we examine the inflammatory response.

The results from our systems biology model appeared to capture the salient features of an independent *in vivo* experiment (that is, an experiment that was not used in the formulation of our model) [37], as shown Fig 3. However, some differences existed between the model results and the experimentally measured quantities. These differences may have arisen because of the model limitations discussed above, or due to experimental constraints of the *in vivo* study. The experimental constraints included the small sample size and the cross-sectional study design in which a different group of people was tested at each time point, making it difficult to infer the time course of cytokine concentrations for a single subject. In addition, the experiments utilized mixed groups of males and females, not accounting for hormonal effects. Uncontrolled hormonal influences in the *in vivo* data may have been responsible for the deviations of experimental data from the model results, which we calculated without hormonal influences for the comparison analysis. Ideally, the results from the present study could be compared to an experiment that separates males and females and controls for female hormone levels, but no such study exists as of yet. The *in vivo* study by Irie et al. appears to be the only study that reports the time course of cytokine concentration in the synovium after injury, and even so, it reports only a subset of the cytokines that we modeled [37].

Irie et al. reported the time course of concentration for IL-6 in addition to the time course for IL-1β, TNF-α, and IL-10 [37]. However, the concentrations for IL-6 in their time course data appear to be two orders of magnitude higher than other reported IL-6 concentrations in the synovium at single time points after ACL injury and in other joint pathologies (see S4 Table), while the concentrations of IL-1β, TNF-α, and IL-10 appear to be consistent with numerical values from comparable measurements (S4 Table). Thus, due to the disagreement in experimental IL-6 concentrations, it is impossible to make a confident comparison of our data. Nevertheless, we note that our results, shown in S1 Fig, seem to quantitatively agree with several other IL-6 measurements from synovial fluid, which we report in S4 Table.

### 4.5 Implications of findings and conclusion

After verification of the model, we performed a preliminary analysis where we considered the effects of sex hormones at single concentrations using the nominal parameter set. The purpose of this analysis was to assess whether sex hormones would have any appreciable effect on inflammation before proceeding to a more thorough analysis in which we varied both the model parameters and the hormonal concentrations. The preliminary analysis revealed that estrogen treatment increased IL-1β and TNF-α and decreased IL-10 compared to testosterone treatment, suggesting that females, who have higher levels of estrogen than males, may be prone to a stronger inflammatory response after injury compared to males. However, females have non-negligible concentrations of testosterone and males have non-negligible concentrations of estrogen, so analysis of isolated hormone effects is inadequate for studying sex differences in the post-injury inflammatory response. Thus, we performed analysis where we simultaneously considered estrogen and testosterone at concentrations relevant for males and females.

In our preliminary analysis of isolated hormones, we also found no discernible change in the inflammatory response when we compared the condition with estrogen alone to the condition with estrogen plus progesterone, suggesting that progesterone was unable to attenuate the effects of estrogen. This result contrasted with our hypothesis that the anti-inflammatory effects of progesterone would be beneficial in the environment of the injured knee. Further, this result was in direct contrast with the findings of other examinations of progesterone effects on inflammation [7, 56]. Specifically, studies of the female reproductive system have shown that progesterone reduces inflammation at the concentrations found locally in the female reproductive system [56]. However, the concentrations in the reproductive tract are substantially higher than those in the synovium (and in our simulations), so it is possible that the concentrations in the synovium were too low to have a meaningful effect. Indeed, the anti-inflammatory effects of progesterone started to become apparent in our model at a concentration of 31.4 pg/mL (see S3 Table). However, because we saw no effect of progesterone at concentrations relevant in the synovium, we did not include it in the subsequent analysis of combined hormones.

Our analysis of combined hormones revealed that a relative abundance of testosterone in males may reduce TNF-α concentrations, since the “Male” condition led to significantly lower TNF-α concentrations compared to both the “Female Peak E” and “Female Low E” conditions. Conversely, estrogen does not appear to have an effect on TNF-α, since no significant difference existed between the “Female Peak E” and “Female Low E” conditions at any time point for the cytokine. The lack of estrogen effect seems to suggest that testosterone is the key hormonal regulator of TNF-α in the present study. This reduction in TNF-α concentration for males may have the potential to improve post-injury outcomes, since the cytokine was recently identified as a marker for early osteoarthritis, a common long-term consequence of ACL injury [57].

Our analysis of combined hormones also revealed that elevated estrogen (“Female Peak E”) seems to exacerbate inflammation compared to lower estrogen (“Female Low E”), even when we include the opposing effects of testosterone at concentrations relevant for females. Indeed, we observed that the “Female Peak E” condition led to significantly elevated IL-1β and MMP-1 and led to significantly reduced IL-10 concentrations compared to “Female Low E” at all time points. This result suggests that the severity of post-injury inflammation may depend not only on the sex of the patient, but also on estrogen concentration at the time of injury for female patients. This result may inform the design of future studies that seek to examine sex differences in post-injury outcomes, since it suggests that inflammation may differ among females, depending on their estrogen levels.

With this quantitative framework, we aimed to shed light on the mechanisms that may cause increased cartilage damage for females after knee injury. Our model results demonstrated that sex hormones could differentially regulate the inflammatory process after an injury and provided a quantitative framework for studying the complexities of the inflammation that may occur after an injury. While the results of this study alone do not provide enough evidence to conclusively assert that estrogen will increase MMP production *in vivo* and, in turn, put females at higher risk of cartilage damage, the results do offer hints toward the mechanism that may underlie poorer post-injury outcomes for females. These hints may help inform the design of future *in vitro* and *in vivo* studies that will further improve our understanding of the factors that may put females at higher risk of damage to the cartilage and poor long-term joint health and may eventually serve as a basis for developing targeted treatments to reduce inflammation and MMPs, particularly for females.

## Supporting information

S1 Text Sample calculations. (DOCX)

S1 Fig Cell, cytokine, MMP, and TIMP concentrations in the absence of hormonal effects for substances in the model that are not depicted in Fig 3. (TIF)

S1 Table Production and decay coefficients. (DOCX)

S2 Table Feedback functions and coefficients. (DOCX)

S3 Table Effect of progesterone on peak TNF concentrations. (DOCX)

S4 Table Experimental Measurements of Synovial Fluid Concentrations of Cytokines, MMPs, and TIMP-1. (DOCX)

S1 Scripts MATLAB Files for all simulations and analysis. (ZIP)
