# Supplementary material for: In silico study of principal sex hormone effects on post-injury synovial inflammatory response
Source: PLoS One. 2018 Dec 31;13(12):e0209582. doi: 10.1371/journal.pone.0209582 (PMC6312367; doi:10.1371/journal.pone.0209582)
Supplement: S1 Text — (DOCX) [file pone.0209582.s001.docx]

**Sample Calculations**

*This procedure has been reported previously [1].*

**1 Production coefficient** **estimation**

- Coefficient of interest: TNF-a production by SFs
- Citation: Huang et al. 2011 [2]
- Table in citation: Table 3 (TNF-a for non-induced SFs)

$$C_{TNF}=9.3 \frac{pg}{mL}$$

$$C_{SF}= 3\times{10}^{5}\frac{cells}{well}$$

Assuming a volume of 2 mL per well in a six well plate,

$$C_{SF}= 1.5\times{10}^{5}\frac{cells}{mL}$$

$$t=24 h$$

$$k_{TGF,SF}=\frac{C_{TGF}}{C_{SF}t}$$

$$k_{TGF,SF}=\frac{0.0093 \frac{ng}{mL}}{1.5\times{10}^{5}\frac{cells}{mL}*24 h}$$

$$\boldsymbol{k}_{\boldsymbol{TGF,SF}}\boldsymbol{=2.58}\boldsymbol{\times}\boldsymbol{10}^{\boldsymbol{-9}}\frac{\boldsymbol{ng}}{\boldsymbol{cell*h}}$$

We note that we had to make two assumptions here. First, we had to assume the volume for a six-well culture plate, which is typically between 1 mL and 2 mL. Second, we assumed that the SFs had not already assumed a pro-inflammatory phenotype (i.e., they were “non-induced”). However, we argue that such assumptions are justified because of our Latin Hypercube Sampling analysis, which varies all estimated parameters around their estimated values to account for the variability that arises from assumptions like these.

**2 Degradation coefficient estimation**

- Coefficient of interest: TNF-a degradation
- Citation: Kaneda et al. 2004 [3]
- Table in citation: Table 2 (TNF-a half-life)

$$t_{1/2}=5 min=0.0833 h$$

$$k_{d,TNF}=\frac{0.693}{t_{1/2}}$$

$$k_{d,TNF}=\frac{0.693}{0.0833 h}$$

$$\boldsymbol{k}_{\boldsymbol{d,TNF}}\boldsymbol{=8.32}\boldsymbol{h}^{\boldsymbol{-1}}$$

**3 Up-regulation feedback parameter estimation**

- Function of interest: TNF-a up-regulation of MMP-9 production by M1
- Citation: Saren et al. 1996 [4]
- Figure in citation: Figure 2
- Data points: (obtained from Saren et al. Fig. 2 using PlotDigitizer © 2000-2015, Joseph A. Huwaldt)

| TNF-a (ng/mL) | MMP-9 (ug/ug DNA) | Normalized MMP-9 |
| --- | --- | --- |
| 0 | 1.218 | 0 |
| 20 | 2.747 | 1.255 |
| 40 | 3.153 | 1.589 |

- Normalization of MMP-9 concentrations:

$$\left\{ C_{MMP9,N} \right\}=\frac{\left\{ C_{MMP9} \right\}-C_{MMP9,min}}{C_{MMP9,min}}$$

where *{C_MMP9,N_}* is the normalized vector of MMP-9 concentrations, *{C_MMP9_}* is the vector of un-normalized MMP-9 concentrations, and *C_MMP9,min_* is the smallest value in the un-normalized vector.

- General form of the up-regulation function

$$a*\frac{C_{TNF}}{1+C_{TNF}}$$

- In the MATLAB curve fitting toolbox, use the vector of TNF-a concentrations as the X-data and the normalized MMP-9 vector as the Y-data. Next, change the fit type from Polynomial to Custom using the dropdown menu. Enter the general form of the equation in the textbox:

$$a*x/(1+x)$$

- In the results box the value of *a* will be listed under coefficients. In this case, *a = 1.531*

4 Down-regulation feedback function parameter estimation

- Function of interest: TNF-a down-regulation of TIMP-1 production by M1
- Citation: Saren et al. 1996 [4]
- Figure in citation: Figure 7 (Data for LPS treatment alone with data for combined LPS+TNF-a treatment)
- Data points: (obtained from Saren et al. Fig. 7 using PlotDigitizer © 2000-2015, Joseph A. Huwaldt)

| TNF-a (ng/mL) | TIMP-1 (ug/ug DNA) | Normalized TIMP-1 |
| --- | --- | --- |
| 0 (LPS only) | 0.181 | 1.00 |
| 2 | 0.071 | 0.392 |
| 20 | 0.071 | 0.392 |
| 40 | 0.071 | 0.392 |

- Normalization of TIMP-1 concentrations:

$$\left\{ C_{TIMP1,N} \right\}=\frac{\left\{ C_{TIMP1} \right\}}{C_{TIMP1,max}}$$

where *{C_TIMP1,N_}* is the normalized vector of TIMP-1 concentrations, *{C_TIMP1_}* is the vector of un-normalized TIMP-1 concentrations, and *C_TIMP1,max_* is the largest value in the un-normalized vector.

- General form of the down-regulation function

$$a*\exp\left( b*C_{TNF} \right)+c$$

- In the MATLAB curve fitting toolbox, use the vector of TNF-a as the X-data and use the normalized TIMP-1 vector as the Y-data. Next change the fit type from Polynomial to Custom using the dropdown menu. Enter the general form of the equation:

$$a*\exp\left( -b*x \right)+c$$

In the results box the value of *a* will be listed under coefficients. In this case, *a = 0.608, b = 3.721, c = 0.392*. Note that the fitted value for *b* can vary slightly. The value listed in Supplementary Table 2 is 3.683, while here it is 3.721. However, these small variations, in general, have very little effect on the model output.

**References**

1. Nagaraja S, Wallqvist A, Reifman J, Mitrophanov AY. Computational approach to characterize causative factors and molecular indicators of chronic wound inflammation. Journal of immunology. 2014;192(4):1824-34.

2. Huang TL, Hsu HC, Yang KC, Lin FH. Hyaluronan up-regulates IL-10 expression in fibroblast-like synoviocytes from patients with tibia plateau fracture. Journal of orthopaedic research : official publication of the Orthopaedic Research Society. 2011;29(4):495-500.

3. Kaneda Y, Tsutsumi Y, Yoshioka Y, Kamada H, Yamamoto Y, Kodaira H, et al. The use of PVP as a polymeric carrier to improve the plasma half-life of drugs. Biomaterials. 2004;25(16):3259-66.

4. Saren P, Welgus HG, Kovanen PT. TNF-alpha and IL-1beta selectively induce expression of 92-kDa gelatinase by human macrophages. Journal of immunology. 1996;157(9):4159-65.
