# Supplementary figures and images for: In silico study of principal sex hormone effects on post-injury synovial inflammatory response
Source: PLoS One. 2018 Dec 31;13(12):e0209582. doi: 10.1371/journal.pone.0209582 (PMC6312367; doi:10.1371/journal.pone.0209582)

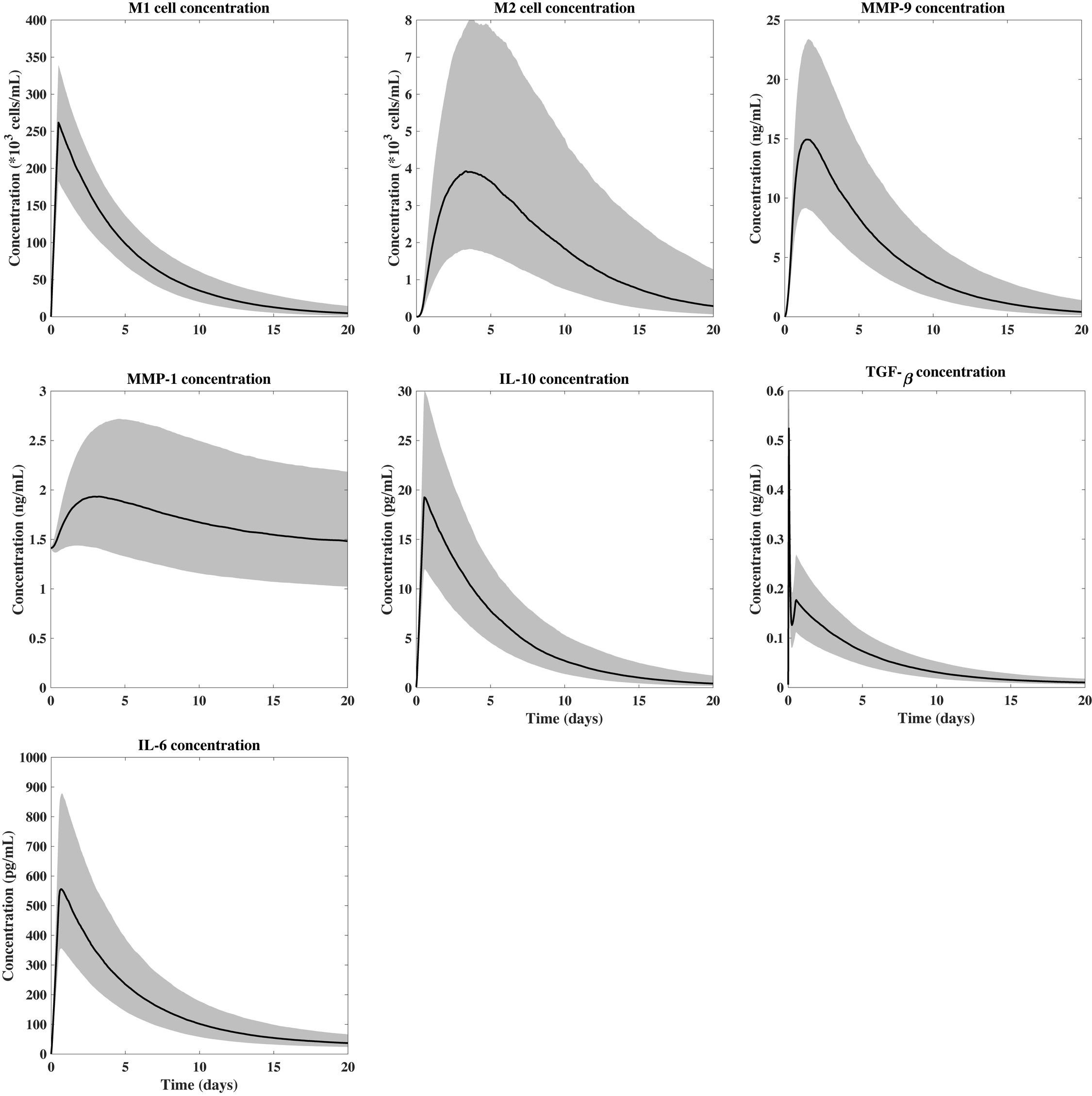

Supplement: S1 Fig — (TIF) [file pone.0209582.s002.tif]
