# Supplementary material for: In silico study of principal sex hormone effects on post-injury synovial inflammatory response
Source: PLoS One. 2018 Dec 31;13(12):e0209582. doi: 10.1371/journal.pone.0209582 (PMC6312367; doi:10.1371/journal.pone.0209582)
Supplement: S1 Table — (DOCX) [file pone.0209582.s003.docx]

| **S1 Table: Production and decay coefficients.** | | | | | |
| --- | --- | --- | --- | --- | --- |
| **Parameter** | **Notation** | **Value** | **Units** | **Sex** | **Citation** |
| Platelet decay | $k_{d,P}$ | $0.69$ | ${hr}^{-1}$ |  | [1]* |
| M1 influx | $k_{M,in}$ | $400$ | ${mL}^{-1}$ |  | [1]* |
| M1/M2 clearance | $k_{M1M2}$ | $8.30*{10}^{-3}$ | ${hr}^{-1}$ |  | [2]* |
| M1 to M2 rate | $k_{d,M}$ | $0.0833$ | ${hr}^{-1}$ |  | Assumed |
| M1 production of IL-1β | $k_{IL1,M1}$ | $1.23*{10}^{-6}$ | $\frac{ng}{cell*hr}$ | N.R. | [3]* |
| M2 production of IL-1β | $k_{IL1,M2}$ | $2.45*{10}^{-7}$ | $\frac{ng}{cell*hr}$ | N.R. | [3]* |
| SF production of IL-1β | $k_{IL1,SF}$ | $1.03*{10}^{-9}$ | $\frac{ng}{cell*hr}$ | M, F | [4] |
| IL-1β decay | $k_{d,IL1}$ | $13.9$ | ${hr}^{-1}$ | M | [5] |
| M1 production of TNF-α | $k_{TNF,M1}$ | $3.46*{10}^{-7}$ | $\frac{ng}{cell*hr}$ | N.R. | [3]* |
| M2 production of TNF-α | $k_{TNF,M2}$ | $4.29*{10}^{-8}$ | $\frac{ng}{cell*hr}$ | N.R. | [3]* |
| SF production of TNF-α | $k_{TNF,SF}$ | $2.58*{10}^{-9}$ | $\frac{ng}{cell*hr}$ | M, F | [4] |
| TNF-α decay | $k_{d,TNF}$ | $8.32$ | ${hr}^{-1}$ | M, F | [6] |
| M1 production of IL-6 | $k_{IL6,M1}$ | $1.18*{10}^{-6}$ | $\frac{ng}{cell*hr}$ | N.R. | [7]* |
| M2 production of IL-6 | $k_{IL6,M2}$ | $1.18*{10}^{-7}$ | $\frac{ng}{cell*hr}$ |  | [1]* |
| SF production of IL-6 | $k_{IL6,SF}$ |  | $\frac{ng}{cell*hr}$ | N.R. | [8] |
| IL-6 decay | $k_{d,IL6}$ | $0.634$ | ${hr}^{-1}$ | N.R. | [9]* |
| M1 production of IL-10 | $k_{IL10,M1}$ | $7.60*{10}^{-8}$ | $\frac{ng}{cell*hr}$ | N.R. | [3]* |
| M2 production of IL-10 | $k_{IL10,M2}$ | $1.55*{10}^{-7}$ | $\frac{ng}{cell*hr}$ | N.R. | [3]* |
| SF production of IL-10 | $k_{IL10,SF}$ | $3.06*{10}^{-10}$ | $\frac{ng}{cell*hr}$ | M, F | [4] |
| IL-10 decay | $k_{d,IL10}$ | $2.079$ | ${hr}^{-1}$ |  | [10] |
| Platelet production of TGF-β | $k_{TGF,P}$ | $1.25*{10}^{-8}$ | $\frac{ng}{cell*hr}$ | N.R. | [11, 12]* |
| M1 production of TGF-β | $k_{TGF,M1}$ | $1.88*{10}^{-6}$ | $\frac{ng}{cell*hr}$ | N.R. | [13]* |
| M2 production of TGF-β | $k_{TGF,M2}$ | $1.60*{10}^{-8}$ | $\frac{ng}{cell*hr}$ | N.R. | [13]* |
| SF production of TGF-β | $k_{TGF,SF}$ | $3.15*{10}^{-8}$ | $\frac{ng}{cell*hr}$ | N.R. | [14] |
| TGF-β decay | $k_{d,TGF}$ | $2.772$ | ${hr}^{-1}$ | N.R. | [15] |
| M1 production of MMP-9 | $k_{MMP9,M1}$ | $6.77*{10}^{-6}$ | $\frac{ng}{cell*hr}$ | M, F | [16] |
| M2 production of MMP-9 | $k_{MMP9,M2}$ | $1.43*{10}^{-5}$ | $\frac{ng}{cell*hr}$ | M, F | [16] |
| MMP-9 decay | $k_{d,MMP9}$ | $0.099$ | ${hr}^{-1}$ |  | [17] |
| M1 production of MMP-1 | $k_{MMP1,M1}$ | $2.00*{10}^{-8}$ | $\frac{ng}{cell*hr}$ | N.R. | [18]* |
| SF production of MMP-1 | $k_{MMP1,SF}$ | $7.24*{10}^{-8}$ | $\frac{ng}{cell*hr}$ | N.R. | [19] |
| MMP-1 decay | $k_{d,MMP1}$ | $0.0257$ | ${hr}^{-1}$ | N.R. | [20] |
| M1 production of TIMP-1 | $k_{TIMP,M1}$ | $3.89*{10}^{-7}$ | $\frac{ng}{cell*hr}$ | M, F | [21] |
| M2 production of TIMP-1 | $k_{TIMP,M2}$ | $2.59*{10}^{-7}$ | $\frac{ng}{cell*hr}$ | M, F | [21] |
| SF production of TIMP-1 | $k_{TIMP,SF}$ | $3.05*{10}^{-4}$ | $\frac{ng}{cell*hr}$ | F | [22] |
| TIMP-1 decay | $k_{d,TIMP}$ | $0.63$ | ${hr}^{-1}$ | F | [23] |
| * Denotes parameters taken directly from [1] | | | | | |

**References**

1. Nagaraja S, Wallqvist A, Reifman J, Mitrophanov AY. Computational approach to characterize causative factors and molecular indicators of chronic wound inflammation. Journal of immunology. 2014;192(4):1824-34. doi: 10.4049/jimmunol.1302481. PubMed PMID: 24453259.

2. Cobbold CA, Sherratt JA. Mathematical modelling of nitric oxide activity in wound healing can explain keloid and hypertrophic scarring. Journal of theoretical biology. 2000;204(2):257-88. doi: 10.1006/jtbi.2000.2012. PubMed PMID: 10887905.

3. Byrne A, Reen DJ. Lipopolysaccharide induces rapid production of IL-10 by monocytes in the presence of apoptotic neutrophils. Journal of immunology. 2002;168(4):1968-77. PubMed PMID: 11823533.

4. Huang TL, Hsu HC, Yang KC, Lin FH. Hyaluronan up-regulates IL-10 expression in fibroblast-like synoviocytes from patients with tibia plateau fracture. Journal of orthopaedic research : official publication of the Orthopaedic Research Society. 2011;29(4):495-500. doi: 10.1002/jor.21261. PubMed PMID: 20957732.

5. Klapproth J, Castell J, Geiger T, Andus T, Heinrich PC. Fate and biological action of human recombinant interleukin 1 beta in the rat in vivo. Eur J Immunol. 1989;19(8):1485-90. doi: 10.1002/eji.1830190821. PubMed PMID: 2476319.

6. Kaneda Y, Tsutsumi Y, Yoshioka Y, Kamada H, Yamamoto Y, Kodaira H, et al. The use of PVP as a polymeric carrier to improve the plasma half-life of drugs. Biomaterials. 2004;25(16):3259-66. doi: 10.1016/j.biomaterials.2003.10.003. PubMed PMID: 14980420.

7. Smythies LE, Sellers M, Clements RH, Mosteller-Barnum M, Meng G, Benjamin WH, et al. Human intestinal macrophages display profound inflammatory anergy despite avid phagocytic and bacteriocidal activity. J Clin Invest. 2005;115(1):66-75. Epub 2005/01/05. doi: 10.1172/JCI19229. PubMed PMID: 15630445; PubMed Central PMCID: PMCPMC539188.

8. Inoue H, Takamori M, Nagata N, Nishikawa T, Oda H, Yamamoto S, et al. An investigation of cell proliferation and soluble mediators induced by interleukin 1beta in human synovial fibroblasts: comparative response in osteoarthritis and rheumatoid arthritis. Inflamm Res. 2001;50(2):65-72. Epub 2001/04/06. doi: 10.1007/s000110050726. PubMed PMID: 11289656.

9. Wong S, Schwartz RC, Pestka JJ. Superinduction of TNF-alpha and IL-6 in macrophages by vomitoxin (deoxynivalenol) modulated by mRNA stabilization. Toxicology. 2001;161(1-2):139-49. Epub 2001/04/11. PubMed PMID: 11295263.

10. Reynolds A, Rubin J, Clermont G, Day J, Vodovotz Y, Bard Ermentrout G. A reduced mathematical model of the acute inflammatory response: I. Derivation of model and analysis of anti-inflammation. Journal of theoretical biology. 2006;242(1):220-36. doi: 10.1016/j.jtbi.2006.02.016. PubMed PMID: 16584750.

11. Grainger DJ, Wakefield L, Bethell HW, Farndale RW, Metcalfe JC. Release and activation of platelet latent TGF-beta in blood clots during dissolution with plasmin. Nat Med. 1995;1(9):932-7. PubMed PMID: 7585220.

12. Wakefield LM, Smith DM, Flanders KC, Sporn MB. Latent transforming growth factor-beta from human platelets. A high molecular weight complex containing precursor sequences. The Journal of biological chemistry. 1988;263(16):7646-54. PubMed PMID: 3163692.

13. Fadok VA, Bratton DL, Konowal A, Freed PW, Westcott JY, Henson PM. Macrophages that have ingested apoptotic cells in vitro inhibit proinflammatory cytokine production through autocrine/paracrine mechanisms involving TGF-beta, PGE2, and PAF. J Clin Invest. 1998;101(4):890-8. doi: 10.1172/JCI1112. PubMed PMID: 9466984; PubMed Central PMCID: PMCPMC508637.

14. Li J, Shao X, Wu L, Feng T, Jin C, Fang M, et al. Honokiol: an effective inhibitor of tumor necrosis factor-alpha-induced up-regulation of inflammatory cytokine and chemokine production in human synovial fibroblasts. Acta Biochim Biophys Sin (Shanghai). 2011;43(5):380-6. doi: 10.1093/abbs/gmr027. PubMed PMID: 21511722.

15. Tarrant JM. Blood cytokines as biomarkers of in vivo toxicity in preclinical safety assessment: considerations for their use. Toxicol Sci. 2010;117(1):4-16. doi: 10.1093/toxsci/kfq134. PubMed PMID: 20447938; PubMed Central PMCID: PMCPMC2923281.

16. Jager NA, Wallis de Vries BM, Hillebrands JL, Harlaar NJ, Tio RA, Slart RH, et al. Distribution of Matrix Metalloproteinases in Human Atherosclerotic Carotid Plaques and Their Production by Smooth Muscle Cells and Macrophage Subsets. Mol Imaging Biol. 2016;18(2):283-91. doi: 10.1007/s11307-015-0882-0. PubMed PMID: 26377769; PubMed Central PMCID: PMCPMC4783451.

17. Saarialho-Kere UK, Welgus HG, Parks WC. Distinct mechanisms regulate interstitial collagenase and 92-kDa gelatinase expression in human monocytic-like cells exposed to bacterial endotoxin. The Journal of biological chemistry. 1993;268(23):17354-61. PubMed PMID: 8394340.

18. Serra R, Al-Saidi AG, Angelov N, Nares S. Suppression of LPS-induced matrix-metalloproteinase responses in macrophages exposed to phenytoin and its metabolite, 5-(p-hydroxyphenyl-), 5-phenylhydantoin. J Inflamm (Lond). 2010;7:48. doi: 10.1186/1476-9255-7-48. PubMed PMID: 20843335; PubMed Central PMCID: PMCPMC2949711.

19. Cha HS, Ahn KS, Jeon CH, Kim J, Song YW, Koh EM. Influence of hypoxia on the expression of matrix metalloproteinase-1, -3 and tissue inhibitor of metalloproteinase-1 in rheumatoid synovial fibroblasts. Clin Exp Rheumatol. 2003;21(5):593-8. PubMed PMID: 14611107.

20. McCachren SS, Greer PK, Niedel JE. Regulation of human synovial fibroblast collagenase messenger RNA by interleukin-1. Arthritis and rheumatism. 1989;32(12):1539-45. PubMed PMID: 2557044.

21. Russell RE, Culpitt SV, DeMatos C, Donnelly L, Smith M, Wiggins J, et al. Release and activity of matrix metalloproteinase-9 and tissue inhibitor of metalloproteinase-1 by alveolar macrophages from patients with chronic obstructive pulmonary disease. Am J Respir Cell Mol Biol. 2002;26(5):602-9. doi: 10.1165/ajrcmb.26.5.4685. PubMed PMID: 11970913.

22. Asano K, Sakai M, Matsuda T, Tanaka H, Fujii K, Hisamitsu T. Suppression of matrix metalloproteinase production from synovial fibroblasts by meloxicam in-vitro. J Pharm Pharmacol. 2006;58(3):359-66. doi: 10.1211/jpp.58.3.0010. PubMed PMID: 16536903.

23. Batra J, Robinson J, Mehner C, Hockla A, Miller E, Radisky DC, et al. PEGylation extends circulation half-life while preserving in vitro and in vivo activity of tissue inhibitor of metalloproteinases-1 (TIMP-1). PloS one. 2012;7(11):e50028. doi: 10.1371/journal.pone.0050028. PubMed PMID: 23185522; PubMed Central PMCID: PMCPMC3502186.
