# Supplementary material for: In silico study of principal sex hormone effects on post-injury synovial inflammatory response
Source: PLoS One. 2018 Dec 31;13(12):e0209582. doi: 10.1371/journal.pone.0209582 (PMC6312367; doi:10.1371/journal.pone.0209582)
Supplement: S2 Table — (DOCX) [file pone.0209582.s004.docx]

| **S2 Table. Feedback functions and coefficients.** | | | | | | | |
| --- | --- | --- | --- | --- | --- | --- | --- |
| **Effector** | **Affected substance** | | **Equation** | **Notation** | **Value** | **Sex** | **Citation** |
| IL-10 effect on | M1 TNF-α | | $f_{IL10,TNF}=a*\exp\left( bC_{IL10} \right)+c$ | $a_{IL10,TNF}$ | $0.467$ | N.R. | [1]* |
|  |  |  |  | $b_{IL10,TNF}$ | $-1.528$ |  |  |
|  |  |  |  | $c_{IL10,TNF}$ | $0.533$ |  |  |
|  | M1 IL-1β | | $f_{IL10,IL1}=a*exp \left( bC_{IL10} \right)+c$ | $a_{IL10,IL1}$ | $0.633$ | N.R. | [1]* |
|  |  |  |  | $b_{IL10,IL1}$ | $-1.794$ |  |  |
|  |  |  |  | $c_{IL10,IL1}$ | $0.367$ |  |  |
|  | M1 IL-6 | | $f_{IL10,IL6}=a*exp \left( bC_{IL10} \right)+c$ | $a_{IL10,IL6}$ | $0.3298$ | N.R. | [1]* |
|  |  |  |  | $b_{IL10,IL6}$ | $-1.189$ |  |  |
|  |  |  |  | $c_{IL10,IL6}$ | $0.6695$ |  |  |
|  | M1 MMP-9 | | $f_{IL10,MMP9}=\exp\left( aC_{IL10} \right)$ | $a_{IL10,MMP9}$ | $-0.0089$ | N.R. | [2] |
|  | M1 TIMP-1 | | $f_{IL10,TIMP}=\exp\left( aC_{IL10} \right)$ | $a_{IL10,TIMP}$ | $-0.0716$ | N.R. | [3] |
|  | M1/M2 transformation | | $f_{M1M2}=\frac{a*C_{IL10}}{1+{a*C}_{IL10}}$ | $a_{M1M2}$ | $0.3213$ | N.R. | [4] |
| TGF-β effect on | M1 TNF-α | | $f_{TGF,TNF}=a*\exp\left( bC_{TGF} \right)+c$ | $a_{TGF,TNF}$ | $0.621$ | N.R. | [5]* |
|  |  |  |  | $b_{TGF,TNF}$ | $-0.831$ |  |  |
|  |  |  |  | $c_{TGF,TNF}$ | $0.447$ |  |  |
|  | M1 IL-1β | | $f_{TGF,IL1}=a*exp \left( bC_{TGF} \right)+c$ | $a_{TGF,IL1}$ | $0.690$ | N.R. | [5]* |
|  |  |  |  | $b_{TGF,IL1}$ | $-20.37$ |  |  |
|  |  |  |  | $c_{TGF,IL1}$ | $0.310$ |  |  |
|  | M1 IL-10 | | $f_{TGF,IL10}=\frac{a*C_{TGF}}{1+a*C_{TGF}}$ | $a_{TGF,IL10}$ | $274.5$ | F | [6]* |
|  | M1 migration | | $f_{TGF,M1}=r_{q1}C_{TGF}^{2}+r_{q2}C_{TGF}$  $C_{TGF}\leq1 pg/mL$ | $r_{q1}$ | $-240.3$ | N.R. | [7]* |
|  |  |  |  | $r_{q2}$ | $298.9$ |  |  |
|  |  |  | $f_{TGF,M1}=r_{l1}C_{TGF}+r_{l2}$  $1 pg/mL<C_{TGF}\leq10 pg/mL$ | $r_{l1}$ | $-0.593$ |  |  |
|  |  |  |  | $r_{l2}$ | $60.59$ |  |  |
| TNF-α effect on | M1 MMP-9 | | $f_{TNF,MMP9}=a*\frac{C_{TNF}}{1+C_{TNF}}$ | $a_{TNF,MMP9}$ | $1.531$ | N.R. | [8] |
|  | M1 TIMP-1 | | $f_{TNF,TIMPa}=a*exp\left( bC_{TNF} \right)+c$ | $a_{TNF,TIMPa}$ | $0.608$ | N.R. | [8] |
|  |  |  |  | $b_{TNF,TIMPa}$ | $-3.683$ |  |  |
|  |  |  |  | $c_{TNF,TIMPa}$ | $0.392$ |  |  |
|  | SF MMP-1 | | $f_{TNF,MMP1}=a*\frac{C_{TNF}}{1+C_{TNF}}$ | $a_{TNF,MMP1}$ | $2.782$ | F | [9] |
|  | SF TIMP-1 | | $f_{TNF,TIMPb}=a*\frac{C_{TNF}}{1+C_{TNF}}$ | $a_{TNF,TIMPb}$ | $0.586$ | F | [9] |
|  | SF IL-1β | | $f_{TNF,IL1}=a*\frac{C_{TNF}}{1+C_{TNF}}$ | $a_{TNF,IL1}$ | $3.078$ | F | [10] |
|  | SF IL-6 | | $f_{TNF,IL6}=a*\frac{C_{TNF}}{1+C_{TNF}}$ | $a_{TNF,IL6}$ | 1.066 | N.R. | [11] |
|  | SF TGF-β | | $f_{TNF,TGF}=a*\frac{C_{TNF}}{1+C_{TNF}}$ | $a_{TNF,TGF}$ | $2.296$ | N.R. | [12] |
|  | M1 migration | | $f_{TNF,M1}=r_{1}C_{TNF}^{2}+r_{2}C_{TNF}$ | $r_{1}$ | $-0.316$ | N.R. | [13]* |
|  |  |  |  | $r_{2}$ | $10.71$ |  |  |
| IL-1β effect on | M1 MMP-9 | | $f_{IL1,MMP9}=a*\frac{C_{IL1}}{1+C_{IL1}}$ | $a_{IL1,MMP9}$ | $4.325$ | N.R. | [8] |
|  | M1 TIMP-1 | | $f_{IL1,TIMPa}=aexp\left( bC_{IL1} \right)+c$ | $a_{IL1,TIMPa}$ | $0.610$ | N.R. | [8] |
|  |  |  |  | $b_{IL1,TIMPa}$ | $-0.233$ |  |  |
|  |  |  |  | $c_{IL1,TIMPa}$ | $0.390$ |  |  |
|  | SF IL-6 | | $f_{IL1,IL6}=a*\frac{C_{IL1}}{1+C_{IL1}}$ | $a_{IL1,IL6}$ | 1.987 | N.R. | [14] |
|  | SF MMP-1 | | $f_{IL1,MMP1}=a*\frac{C_{IL1}}{1+C_{IL1}}$ | $a_{IL1,MMP1}$ | $46.76$ | N.R. | [15] |
|  | SF TIMP-1 | | $f_{IL1,TIMPb}=a*\frac{C_{IL1}}{1+C_{IL1}}$ | $a_{IL1,TIMPb}$ | $0.237$ | M, F | [16] |
|  | SF TNF-α | | $f_{IL1,TNF}=a*\frac{C_{IL1}}{1+C_{IL1}}$ | $a_{IL1,TNF}$ | $2.903$ | M, F | [16] |
|  | SF IL-1β | | $f_{IL1,IL1}=a*\frac{C_{IL1}}{1+C_{IL1}}$ | $a_{IL1,IL1}$ | $10.11$ | M, F | [16] |
| IL-6 effect on | M1 IL-1β | | $f_{IL6,IL1}=\frac{a*C_{IL6}}{a+{C_{IL6}}^{b}}$ | $a_{IL6,IL1}$  $b_{IL6,IL1}$ | $4.459$  0.1571 | N.R. | [17]* |
|  | M1 TNF-α | | $f_{IL6,TNF}=\frac{a*C_{IL6}}{a+{C_{IL6}}^{b}}$ | $a_{IL6,TNF}$  $b_{IL6,TNF}$ | $4.488$  0.1541 | N.R. | [17]* |
|  | M1 IL-10 | | $f_{IL6,IL10}=\frac{a*C_{IL6}}{1+{a*C}_{IL6}}$ | $a_{IL6,IL10}$ | 0.1424 | N.R. | [2] |
|  | SF TIMP-1 | | $f_{IL6,TIMP1}=a*\frac{C_{IL6}}{1+C_{IL6}}$ | $a_{IL6,TIMP1}$ | 0.2753 | N.R. | [18] |
| E effect on | M1 IL-1β | | $f_{E2,IL1}=a*\frac{C_{E2}}{1+C_{E2}}$ | $a_{E2,IL1}$ | $5.337$ | F | [19] |
|  | M1 IL-6 | | $f_{E2,IL6}=exp\left( a*C_{E2} \right)$ | $a_{E2,IL6}$ | $-0.0331$ | M | [20] |
|  | M1 IL-10 | | $f_{E2,IL10}=exp\left( a*C_{E2} \right)$ | $a_{E2,IL10}$ | $-0.139$ | N.R. | [21] |
| T effect on | M1 TNF-α | | $f_{T,TNF}=exp(a*C_{T})$ | $a_{T,TNF}$ | $-0.0742$ | N.R. | [21] |
|  | M1 IL-10 | | $f_{T,IL10}=a*\frac{C_{T}}{1+C_{T}}$ | $a_{T,IL10}$ | $0.522$ | N.R. | [21] |
| P effect on | M1 TNF-α | | $f_{P,TNF}=exp(a*C_{P})$ | $a_{P,TNF}$ | $-0.0478$ | N.R. | [22] |
|  | M1 IL-6 | | $f_{P,IL6}=exp(a*C_{P})$ | $a_{P,IL6}$ | $-0.1083$ |  | [23] |
| * Denotes parameters taken directly from [24] | | | | | | | |
|  | |  |  |  |  |  |  |

**References**

1. Thomassen MJ, Divis LT, Fisher CJ. Regulation of human alveolar macrophage inflammatory cytokine production by interleukin-10. Clin Immunol Immunopathol. 1996;80(3 Pt 1):321-4. PubMed PMID: 8811054.

2. Kothari P, Pestana R, Mesraoua R, Elchaki R, Khan KM, Dannenberg AJ, et al. IL-6-mediated induction of matrix metalloproteinase-9 is modulated by JAK-dependent IL-10 expression in macrophages. Journal of immunology. 2014;192(1):349-57. Epub 2013/11/29. doi: 10.4049/jimmunol.1301906. PubMed PMID: 24285838; PubMed Central PMCID: PMCPMC3872272.

3. Jovanovic DV, Di Battista JA, Martel-Pelletier J, Reboul P, He Y, Jolicoeur FC, et al. Modulation of TIMP-1 synthesis by antiinflammatory cytokines and prostaglandin E2 in interleukin 17 stimulated human monocytes/macrophages. The Journal of rheumatology. 2001;28(4):712-8. PubMed PMID: 11327240.

4. Kuwata H, Watanabe Y, Miyoshi H, Yamamoto M, Kaisho T, Takeda K, et al. IL-10-inducible Bcl-3 negatively regulates LPS-induced TNF-alpha production in macrophages. Blood. 2003;102(12):4123-9. doi: 10.1182/blood-2003-04-1228. PubMed PMID: 12907458.

5. Chantry D, Turner M, Abney E, Feldmann M. Modulation of cytokine production by transforming growth factor-beta. Journal of immunology. 1989;142(12):4295-300. PubMed PMID: 2542408.

6. Maeda H, Kuwahara H, Ichimura Y, Ohtsuki M, Kurakata S, Shiraishi A. TGF-beta enhances macrophage ability to produce IL-10 in normal and tumor-bearing mice. Journal of immunology. 1995;155(10):4926-32. PubMed PMID: 7594497.

7. Wahl SM, Hunt DA, Wakefield LM, McCartney-Francis N, Wahl LM, Roberts AB, et al. Transforming growth factor type beta induces monocyte chemotaxis and growth factor production. Proceedings of the National Academy of Sciences of the United States of America. 1987;84(16):5788-92. PubMed PMID: 2886992; PubMed Central PMCID: PMCPMC298948.

8. Saren P, Welgus HG, Kovanen PT. TNF-alpha and IL-1beta selectively induce expression of 92-kDa gelatinase by human macrophages. Journal of immunology. 1996;157(9):4159-65. PubMed PMID: 8892653.

9. Asano K, Sakai M, Matsuda T, Tanaka H, Fujii K, Hisamitsu T. Suppression of matrix metalloproteinase production from synovial fibroblasts by meloxicam in-vitro. J Pharm Pharmacol. 2006;58(3):359-66. doi: 10.1211/jpp.58.3.0010. PubMed PMID: 16536903.

10. Ganesan K, Balachandran C, Manohar BM, Puvanakrishnan R. Effects of testosterone, estrogen and progesterone on TNF-alpha mediated cellular damage in rat arthritic synovial fibroblasts. Rheumatology international. 2012;32(10):3181-8. doi: 10.1007/s00296-011-2146-x. PubMed PMID: 21960045.

11. Mrosewski I, Jork N, Gorte K, Conrad C, Wiegand E, Kohl B, et al. Regulation of osteoarthritis-associated key mediators by TNFalpha and IL-10: effects of IL-10 overexpression in human synovial fibroblasts and a synovial cell line. Cell and tissue research. 2014;357(1):207-23. Epub 2014/05/13. doi: 10.1007/s00441-014-1868-y. PubMed PMID: 24816983.

12. Li J, Shao X, Wu L, Feng T, Jin C, Fang M, et al. Honokiol: an effective inhibitor of tumor necrosis factor-alpha-induced up-regulation of inflammatory cytokine and chemokine production in human synovial fibroblasts. Acta Biochim Biophys Sin (Shanghai). 2011;43(5):380-6. doi: 10.1093/abbs/gmr027. PubMed PMID: 21511722.

13. Pai R, Ha H, Kirschenbaum MA, Kamanna VS. Role of tumor necrosis factor-alpha on mesangial cell MCP-1 expression and monocyte migration: mechanisms mediated by signal transduction. Journal of the American Society of Nephrology : JASN. 1996;7(6):914-23. PubMed PMID: 8793801.

14. Inoue H, Takamori M, Nagata N, Nishikawa T, Oda H, Yamamoto S, et al. An investigation of cell proliferation and soluble mediators induced by interleukin 1beta in human synovial fibroblasts: comparative response in osteoarthritis and rheumatoid arthritis. Inflamm Res. 2001;50(2):65-72. Epub 2001/04/06. doi: 10.1007/s000110050726. PubMed PMID: 11289656.

15. Yorifuji M, Sawaji Y, Endo K, Kosaka T, Yamamoto K. Limited efficacy of COX-2 inhibitors on nerve growth factor and metalloproteinases expressions in human synovial fibroblasts. J Orthop Sci. 2016;21(3):381-8. doi: 10.1016/j.jos.2016.01.004. PubMed PMID: 26876621.

16. Huang TL, Hsu HC, Yang KC, Lin FH. Hyaluronan up-regulates IL-10 expression in fibroblast-like synoviocytes from patients with tibia plateau fracture. Journal of orthopaedic research : official publication of the Orthopaedic Research Society. 2011;29(4):495-500. doi: 10.1002/jor.21261. PubMed PMID: 20957732.

17. Schindler R, Mancilla J, Endres S, Ghorbani R, Clark SC, Dinarello CA. Correlations and interactions in the production of interleukin-6 (IL-6), IL-1, and tumor necrosis factor (TNF) in human blood mononuclear cells: IL-6 suppresses IL-1 and TNF. Blood. 1990;75(1):40-7. Epub 1990/01/01. PubMed PMID: 2294996.

18. Silacci P, Dayer JM, Desgeorges A, Peter R, Manueddu C, Guerne PA. Interleukin (IL)-6 and its soluble receptor induce TIMP-1 expression in synoviocytes and chondrocytes, and block IL-1-induced collagenolytic activity. The Journal of biological chemistry. 1998;273(22):13625-9. Epub 1998/06/05. PubMed PMID: 9593700.

19. Calippe B, Douin-Echinard V, Delpy L, Laffargue M, Lelu K, Krust A, et al. 17Beta-estradiol promotes TLR4-triggered proinflammatory mediator production through direct estrogen receptor alpha signaling in macrophages in vivo. Journal of immunology. 2010;185(2):1169-76. doi: 10.4049/jimmunol.0902383. PubMed PMID: 20554954.

20. Liu L, Zhao Y, Xie K, Sun X, Jiang L, Gao Y, et al. Estrogen inhibits LPS-induced IL-6 production in macrophages partially via the nongenomic pathway. Immunol Invest. 2014;43(7):693-704. Epub 2014/06/25. doi: 10.3109/08820139.2014.917095. PubMed PMID: 24960169.

21. D'Agostino P, Milano S, Barbera C, Di Bella G, La Rosa M, Ferlazzo V, et al. Sex hormones modulate inflammatory mediators produced by macrophages. Annals of the New York Academy of Sciences. 1999;876:426-9. PubMed PMID: 10415638.

22. Lei B, Mace B, Dawson HN, Warner DS, Laskowitz DT, James ML. Anti-inflammatory effects of progesterone in lipopolysaccharide-stimulated BV-2 microglia. PloS one. 2014;9(7):e103969. doi: 10.1371/journal.pone.0103969. PubMed PMID: 25080336; PubMed Central PMCID: PMC4117574.

23. Sun Y, Cai J, Ma F, Lu P, Huang H, Zhou J. miR-155 mediates suppressive effect of progesterone on TLR3, TLR4-triggered immune response. Immunology letters. 2012;146(1-2):25-30. Epub 2012/05/02. doi: 10.1016/j.imlet.2012.04.007. PubMed PMID: 22546503.

24. Nagaraja S, Wallqvist A, Reifman J, Mitrophanov AY. Computational approach to characterize causative factors and molecular indicators of chronic wound inflammation. Journal of immunology. 2014;192(4):1824-34. doi: 10.4049/jimmunol.1302481. PubMed PMID: 24453259.
