# Supplementary material for: In silico study of principal sex hormone effects on post-injury synovial inflammatory response
Source: PLoS One. 2018 Dec 31;13(12):e0209582. doi: 10.1371/journal.pone.0209582 (PMC6312367; doi:10.1371/journal.pone.0209582)
Supplement: S3 Table — (DOCX) [file pone.0209582.s005.docx]

| **S3 Table: Effect of progesterone on peak TNF concentrations.** | |
| --- | --- |
| Progesterone Concentration (ng/mL) | Peak TNF-a Concentration (pg/mL) |
| 1.4 | 10.77 |
| 3.14 | 10.77 |
| 31.4 | 10.76 |
| 314 | 10.62 |
| 3140 | 9.29 |
