# Supplementary material for: In silico study of principal sex hormone effects on post-injury synovial inflammatory response
Source: PLoS One. 2018 Dec 31;13(12):e0209582. doi: 10.1371/journal.pone.0209582 (PMC6312367; doi:10.1371/journal.pone.0209582)
Supplement: S4 Table — (DOCX) [file pone.0209582.s006.docx]

| **S4 Table.** **Experimental Measurements of Synovial Fluid Concentrations of Cytokines, MMPs, and TIMP-1.** The concentrations of substances in healthy knee joints are shown in **bold** type. These concentrations represent the physiological “initial conditions” prior to injury. | | | | | |
| --- | --- | --- | --- | --- | --- |
| **Substance** | **Average (pg/mL)** | **Error (pg/mL)** | **Condition** | **Number of Females / Total Participants** | **Citation** |
| IL-1β | 7.8 | 3.7 | arthroscopy adolescent (<18 yo) | 7 of 9 | [1] |
|  | 10.1 | 9.5 | arthroscopy adult (>=18 yo) | 11 of 40 |  |
|  | 15.3 | 6.9 | acute acl tear | 0 of 48 | [2] |
|  | 24.9 | 7.5 | acute acl tear + meniscal damage | 0 of 48 |  |
|  | 15.6 | 9.8 | early sub-acute acl tear | 0 of 48 |  |
|  | 5.9 | 2.5 | early sub-acute acl tear +meniscal damage | 0 of 48 |  |
|  | 11 | 3.4 | late sub-acute acl tear | 0 of 48 |  |
|  | 13.1 | 7.3 | late sub-acute acl tear +meniscus damage | 0 of 48 |  |
|  | 14.2 | 10.8 | chronic acl tear | 0 of 48 |  |
|  | 9.5 | 3 | chronic acl tear +meniscal damage | 0 of 48 |  |
|  | 1.2 | 1.6 | OA, pre-TKA | 16 of 28 | [3] |
|  | **1** | **2** | **healthy** | **n.r.** | [4] |
|  | 15 | 18 | cartilage defect | n.r. |  |
|  | 8 | 16 | OA | n.r. |  |
| TNF-α | 0.87 | 0.58 | acute acl tear | 0 of 48 | [2] |
|  | 2.7 | 1.2 | acute acl tear + meniscal damage | 0 of 48 |  |
|  | 4.6 | 3.2 | early sub-acute acl tear | 0 of 48 |  |
|  | 5.6 | 1.7 | early sub-acute acl tear +meniscal damage | 0 of 48 |  |
|  | 3.1 | 1.3 | late sub-acute acl tear | 0 of 48 |  |
|  | 3 | 0.4 | late sub-acute acl tear +meniscus damage | 0 of 48 |  |
|  | 6.3 | 2.8 | chronic acl tear | 0 of 48 |  |
|  | 6.7 | 2 | chronic acl tear +meniscal damage | 0 of 48 |  |
|  | 2.7 | 1.2 | OA, pre-TKA | 16 of 28 | [3] |
|  | **0** | **0** | **healthy** | **n.r.** | [4] |
|  | 2 | 8 | cartilage defect | n.r. |  |
|  | 4 | 20 | OA | n.r. |  |
| IL-10 | 29.14 | 1.8 | acute acl tear | 0 of 48 | [2] |
|  | 28.3 | 3.4 | acute acl tear + meniscal damage | 0 of 48 |  |
|  | 36 | 21.9 | early sub-acute acl tear | 0 of 48 |  |
|  | 16.2 | 4 | early sub-acute acl tear +meniscal damage | 0 of 48 |  |
|  | 7.5 | 1.9 | late sub-acute acl tear | 0 of 48 |  |
|  | 10.8 | 1.7 | late sub-acute acl tear +meniscus damage | 0 of 48 |  |
|  | 3.3 | 3.3 | chronic acl tear | 0 of 48 |  |
|  | 5.5 | 2.8 | chronic acl tear +meniscal damage | 0 of 48 |  |
|  | **1** | **6** | **healthy** | **n.r.** | [4] |
|  | 0 | 0 | cartilage defect | n.r. |  |
|  | 9 | 35 | OA | n.r. |  |
|  | 0 | 0 | TMD | 24 of 31 | [5] |
| TGF-β | 1970 | 530 | healthy knee | 6 of 12 | [6] |
|  | 2320 | 320 | TKA | 17 of 28 |  |
|  | 100.4 | 109.3 | TMD | 24 of 31 | [5] |
|  | 0 | 0 | Healthy TMJ | 4 of 7 |  |
| IL-6 | 576.6 | 166.4 | acute acl tear + meniscal damage | 0 of 48 | [2] |
|  | 504.4 | 247.8 | early sub-acute acl tear | 0 of 48 |  |
|  | 388.3 | 110.8 | early sub-acute acl tear +meniscal damage | 0 of 48 |  |
|  | 24.1 | 8.0 | late sub-acute acl tear | 0 of 48 |  |
|  | 298.1 | 141.1 | late sub-acute acl tear +meniscus damage | 0 of 48 |  |
|  | 193.4 | 110.7 | chronic acl tear | 0 of 48 |  |
|  | 114.9 | 72.5 | OA, pre-TKA | 16 of 28 | [3] |
|  | **64** | **120** | **healthy** | **n.r** | [4] |
|  | 261 | 385 | cartilage defect | n.r. |  |
|  | 396 | 508 | OA | n.r. |  |
|  | 88.8 | 32.3 | healthy knee | 6 of 12 | [6] |
|  | 281.2 | 48.3 | TKA | 17 of 28 |  |
|  | 38.2 | 115.9 | < 6 mo. post ACL | 29 of 67 | [7] |
| MMP-9 | 110 | 220.1 | OA, pre-TKA | 16 of 28 | [3] |
|  | 6520 | n.r. | injured ankle | 14 of 45 | [8] |
|  | 960 | n.r. | un-injured ankle | 14 of 45 |  |
|  | 609 | 290 | OA | n.r. | [9] |
|  | **9511** | **n.r.** | **healthy** | **11 of 25** | [10] |
|  | 11007 | n.r. | early OA | 1 of 12 |  |
|  | 53391 | n.r. | OA | 9 of 17 |  |
|  | 0 | 0 | < 6 mo. post ACL | 29 of 67 | [7] |
| MMP-1 | 3890 | n.r. | injured ankle | 14 of 45 | [8] |
|  | 370 | n.r. | un-injured ankle | 14 of 45 |  |
|  | 549200 | 273100 | OA | n.r. | [9] |
|  | **3729** | **n.r.** | **healthy** | **11 of 25** | [10] |
|  | 8982 | n.r. | early OA | 1 of 12 |  |
|  | 53563 | n.r. | OA | 9 of 17 |  |
|  | 4928 | 6112 | < 6mo. post ACL | 29 of 67 | [7] |
| TIMP-1 | **123925** | **n.r.** | **healthy** | **11 of 25** | [10] |
|  | 115567 | n.r. | early OA | 1 of 12 |  |
|  | 707562 | n.r. | OA | 9 of 17 |  |
|  | 1841461 | 9405495 | < 6 mo. post ACL | 29 of 67 | [7] |

**References**

1. Schmal H, Henkelmann R, Mehlhorn AT, Reising K, Bode G, Sudkamp NP, et al. Synovial cytokine expression in ankle osteoarthritis depends on age and stage. Knee surgery, sports traumatology, arthroscopy : official journal of the ESSKA. 2015;23(5):1359-67. doi: 10.1007/s00167-013-2719-1. PubMed PMID: 24141892.

2. Bigoni M, Sacerdote P, Turati M, Franchi S, Gandolla M, Gaddi D, et al. Acute and late changes in intraarticular cytokine levels following anterior cruciate ligament injury. Journal of orthopaedic research : official publication of the Orthopaedic Research Society. 2013;31(2):315-21. doi: 10.1002/jor.22208. PubMed PMID: 22886741.

3. Gandhi R, Santone D, Takahashi M, Dessouki O, Mahomed NN. Inflammatory predictors of ongoing pain 2 years following knee replacement surgery. Knee. 2013;20(5):316-8. doi: 10.1016/j.knee.2012.10.015. PubMed PMID: 23157967.

4. Tsuchida AI, Beekhuizen M, t Hart MC, Radstake TR, Dhert WJ, Saris DB, et al. Cytokine profiles in the joint depend on pathology, but are different between synovial fluid, cartilage tissue and cultured chondrocytes. Arthritis research & therapy. 2014;16(5):441. doi: 10.1186/s13075-014-0441-0. PubMed PMID: 25256035; PubMed Central PMCID: PMCPMC4201683.

5. Fang PK, Ma XC, Ma DL, Fu KY. Determination of interleukin-1 receptor antagonist, interleukin-10, and transforming growth factor-beta1 in synovial fluid aspirates of patients with temporomandibular disorders. J Oral Maxillofac Surg. 1999;57(8):922-8; discussion 8-9. PubMed PMID: 10437719.

6. Regan EA, Bowler RP, Crapo JD. Joint fluid antioxidants are decreased in osteoarthritic joints compared to joints with macroscopically intact cartilage and subacute injury. Osteoarthritis and cartilage / OARS, Osteoarthritis Research Society. 2008;16(4):515-21. doi: 10.1016/j.joca.2007.09.001. PubMed PMID: 18203633.

7. Tourville TW, Poynter ME, DeSarno MJ, Struglics A, Beynnon BD. Relationship between synovial fluid ARGS-aggrecan fragments, cytokines, MMPs, and TIMPs following acute ACL injury: A cross-sectional study. Journal of orthopaedic research : official publication of the Orthopaedic Research Society. 2015;33(12):1796-803. doi: 10.1002/jor.22961. PubMed PMID: 26123869.

8. Haller JM, Swearingen CA, Partridge D, McFadden M, Thirunavukkarasu K, Higgins TF. Intraarticular Matrix Metalloproteinases and Aggrecan Degradation Are Elevated After Articular Fracture. Clin Orthop Relat Res. 2015;473(10):3280-8. doi: 10.1007/s11999-015-4441-4. PubMed PMID: 26162411; PubMed Central PMCID: PMCPMC4562930.

9. Kim KS, Choi HM, Lee YA, Choi IA, Lee SH, Hong SJ, et al. Expression levels and association of gelatinases MMP-2 and MMP-9 and collagenases MMP-1 and MMP-13 with VEGF in synovial fluid of patients with arthritis. Rheumatology international. 2011;31(4):543-7. doi: 10.1007/s00296-010-1592-1. PubMed PMID: 20665024.

10. Heard BJ, Martin L, Rattner JB, Frank CB, Hart DA, Krawetz R. Matrix metalloproteinase protein expression profiles cannot distinguish between normal and early osteoarthritic synovial fluid. BMC Musculoskelet Disord. 2012;13:126. doi: 10.1186/1471-2474-13-126. PubMed PMID: 22824140; PubMed Central PMCID: PMCPMC3532375.
