# Supplementary material for: In silico study of principal sex hormone effects on post-injury synovial inflammatory response
Source: PLoS One. 2018 Dec 31;13(12):e0209582. doi: 10.1371/journal.pone.0209582 (PMC6312367; doi:10.1371/journal.pone.0209582)
Supplement: S1 Scripts — (ZIP) [file pone.0209582.s007.zip › ScriptFiles/README.docx]

README

| **Scripts used to generate results** | |
| --- | --- |
| main_script.m | Script used to specify different simulation conditions and obtain output data |
| ode_ftn.m | Function that solves the differential equations |
| cytokine_hist.m | Function that sets the initial conditions for the differential equations and specifies the trigger of the inflammatory process |
| all_feedback_ftns.m | Function to calculate the values of all the feedback functions during every step of the ODE solver code |
| rate_coeffs.m | Contains rate coefficients for the differential equations |
| all_parameter_fits.m | Contains the estimates for all fitted parameters. Also includes the raw data extracted from the literature that was used to fit each parameter (citations are noted in the comments) |
| lhs_script_general.m | Function used to vary the simulation parameters with Latin Hypercube Sampling and obtain median and IQR of all the different outputs. |
| lhs_script_ks_and_et.m | Function to simultaneously vary the concentrations of sex hormones and the simulation parameters with Latin Hypercube Sampling and obtain median and IQR of all the different outputs. |
| Ks_mod.m | Function used to generate perturbed parameters during Latin Hypercube Sampling |
| **Scripts used to generate plots** | |
| ciplot_mod.m | Function modified from a freely available code by Raymond Reynolds that plots figures with confidence bands |
| plot_concentrations.m | Function used to generate figures without concentration bands (e.g., Figure 3) |
| plot_concentration_bands.m | Function used to generate figures with concentration bands |
| steady_hormone_plots_ept.m | Script used to generate plots in Figure 3 |
| male_fem_vary_plots.m | Script used to generate plots in Figure 4 |
| compare_to_Irie.m | Script used to generate plots in Figure 2 |
| **Other files** | |
| statistical_comparisons_hormones.m | Script used to perform Kruskal-Wallis and Mann-Whitney U tests between groups at different time points in the analysis of combined estrogen and testosterone |
| hormone_data/normalized_time.csv | Data file used to evenly space time increments |

**Instructions for generating plots found in the paper**

Figure 1: (this is a diagram created in Adobe Illustrator, so it cannot be generated with the code)

Figure 2: (diagram created in MS PowerPoint)

Figure 3 and Supplemental Figure 1:

- Clear the workspace (type “clear all” in the command window)
- Set the variable lhs_ks = 1 (line 70 in main_script.m)
- Set e2_only = 0; e2_plus_p = 0; t_only = 0; vary_et_female = 0; vary_et_male = 0; (lines 83, 84, 85, 94, and 95, respectively, in main_script.m)
- Run main_script.m
  - This may take several hours
  - This will automatically generate the plots in Supplemental Figure 1
- Without clearing the variables, run compare_to_Irie.m

Figure 4:

- Clear the workspace (type “clear all” in the command window)
- Set the variables e2_only = 1; e2_plus_p = 1; t_only = 1; (lines 83, 84, 85 in main_script.m)
- Set lhs_ks = 0; vary_et_female = 0; vary_et_male = 0; (lines 74, 94, and 95, respectively, in main_script.m)
- Run main_script.m
- Without clearing the variables, run steady_hormones_ept_plots.m

Figure 5 and statistical analysis:

- Clear the workspace (type “clear all” in the command window)
- Set the variable vary_et_female = 1; vary_et_male = 1; (lines 90 and 91 in main_script.m)
- Set lhs_ks = 0; e2_only = 0; e2_plus_p = 0; t_only = 0; (lines 70,83, 84, and 85, respectively, in main_script.m)
- Run main_script.m
  - This may take several hours
- Without clearing the variables, run male_fem_vary_plots.m
- Without clearing the variables, run statistical_comparisons_hormones.m
